# Intranasal delivery of a rationally attenuated SARS-CoV-2 is immunogenic and protective in Syrian hamsters

**DOI:** 10.1038/s41467-022-34571-4

**Published:** 2022-11-10

**Authors:** Shufeng Liu, Charles B. Stauft, Prabhuanand Selvaraj, Prabha Chandrasekaran, Felice D’Agnillo, Chao-Kai Chou, Wells W. Wu, Christopher Z. Lien, Clement A. Meseda, Cyntia L. Pedro, Matthew F. Starost, Jerry P. Weir, Tony T. Wang

**Affiliations:** 1 Division of Viral Products, Center for Biologics Evaluation and Research, Food and Drug Administration, Silver Spring, MD USA; 2 Laboratory of Clinical Investigation, National Institutes of Aging, National Institutes of Health, Baltimore, USA; 3 Laboratory of Biochemistry and Vascular Biology, Center for Biologics Evaluation and Research, Food and Drug Administration, Silver Spring, MD USA; 4 Facility for Biotechnology Resources, Center for Biologics Evaluation and Research, Food and Drug Administration, Silver Spring, MD USA; 5 Division of Veterinary Resources, Diagnostic and Research Services Branch, National Institutes of Health, Rockville Pike, USA

**Keywords:** Live attenuated vaccines, SARS-CoV-2

## Abstract

Few live attenuated severe acute respiratory syndrome coronavirus 2 (SARS-CoV-2) vaccines are in pre-clinical or clinical development. We seek to attenuate SARS-CoV-2 (isolate WA1/2020) by removing the polybasic insert within the spike protein and the open reading frames (ORFs) 6–8, and by introducing mutations that abolish non-structural protein 1 (Nsp1)-mediated toxicity. The derived virus (WA1-ΔPRRA-ΔORF6-8-Nsp1^K164A/H165A^) replicates to 100- to 1000-fold-lower titers than the ancestral virus and induces little lung pathology in both K18-human ACE2 (hACE2) transgenic mice and Syrian hamsters. Immunofluorescence and transcriptomic analyses of infected hamsters confirm that three-pronged genetic modifications attenuate the proinflammatory pathways more than the removal of the polybasic cleavage site alone. Finally, intranasal administration of just 100 PFU of the WA1-ΔPRRA-ΔORF6-8-Nsp1^K164A/H165A^ elicits robust antibody responses in Syrian hamsters and protects against SARS-CoV-2-induced weight loss and pneumonia. As a proof-of-concept study, we demonstrate that live but sufficiently attenuated SARS-CoV-2 vaccines may be attainable by rational design.

## Abstract (web summary)

Examples of effective live attenuated viral vaccines include the measles, mumps and rubella vaccine, as well as the chickenpox vaccine. In this work, the authors engineer a live attenuated SARS-CoV-2 virus that demonstrates attenuation in numerous in vivo models, and protection in hamsters upon challenge.

## Introduction

The rapid development of multiple vaccines has afforded mankind powerful tools to curb the severe coronavirus disease 2019 (COVID-19) pandemic. Among the eleven vaccines granted emergency use by the World Health Organization (WHO), seven of them, including the Pfizer and Moderna mRNA vaccines, three adenovirus-vector-based vaccines (AstraZeneca/Oxford, AstraZeneca/Serum Institute of India, J&J), and two protein-based vaccines (Novavax and Covovax with Novavax formulation), all express SARS-CoV-2 spike protein as the immunogen. The other three inactivated, whole virus vaccines (SinoPharm, Sinovac, and Bharat Biotech) appear to be less immunogenic in inducing neutralizing antibodies^1–3^. The efficacy of existing vaccines in preventing symptomatic infections, especially against new variants of concern, declines considerably over a period of six months^4–11^. The stringent storage conditions and requirement of medical supplies to administer the vaccines put additional constraints on the worldwide distribution of some vaccines. For these reasons, new vaccines that are potent, broadly protective, and elicit durable immunity, as well as being easy to administer, store, and transport must be continuously pursued.

Live attenuated viral vaccines (LAV) utilize a living but weakened virus as immunogen, and there are many examples of effective LAVs including the measles, mumps, and rubella vaccine, oral polio vaccine, yellow fever virus vaccine, chickenpox vaccine, and one influenza vaccine. LAV causes a real, but often asymptomatic infection in vaccinees, and hence usually elicits both humoral and cellular immune responses. In addition, an intranasally administered LAV will not only avoid needle sticks but may be more effective in eliciting immunity at the mucosal membrane. The latter is especially desirable for prevention of COVID-19 because the human upper respiratory airway tends to be less protected by existing vaccines that are administered intramuscularly^12^. An obvious obstacle to the development of LAVs against SARS-CoV-2 is the safety of the vaccine virus. Multi-layer attenuation of pathogenesis is expected to ensure a LAV does not revert back to virulence. To date, the only LAV that is in clinical development against COVID-19 is a genetically recoded virus with a segment of the viral spike protein being codon-pair deoptimized (clinicaltrials.gov Identifier: NCT04619628)^13^.

It is worth noting that SARS-CoV-2 attenuation does occur naturally and in cell culture. The most common mechanism of attenuation is the loss of the polybasic insert (*PRRA*) or the furin cleavage site^14,15^, which impairs the ability for the virus to infect the lung^16,17^. SARS-CoV-2 is also known to encode several powerful viral proteins that subvert the host innate immunity, especially interferons and interferon-stimulated genes (ISGs), and hence promote infection^18^. Numerous studies have reported that non-structural protein 1 (Nsp1), and accessory proteins ORF6, 7, and 8 are potent interferon (IFN) antagonists^19–23^. Nsp1 is also cytopathic in cells of human lung origin^24^. Clinical isolates containing a 382-nucleotide deletion in ORF8 seem to be associated with a milder infection^25^. Taken together, there is a strong rationale to explore further development of attenuated SARS-CoV-2 vaccines and sufficient information currently available to provide a basis for the rational attenuation.

Here, we demonstrate a generic strategy to attenuate SARS-CoV-2 through reverse genetics. The derived virus (WA1-ΔPRRA-ΔORF6-8-Nsp1^K164A/H165A^) replicates to 100- to 1000-fold-lower titers than the ancestral virus, induces robust antibody responses in Syrian hamsters and protects against SARS-CoV-2 challenge.

## Results

### Rational Attenuation of SARS-CoV-2

**Fig. 1:**
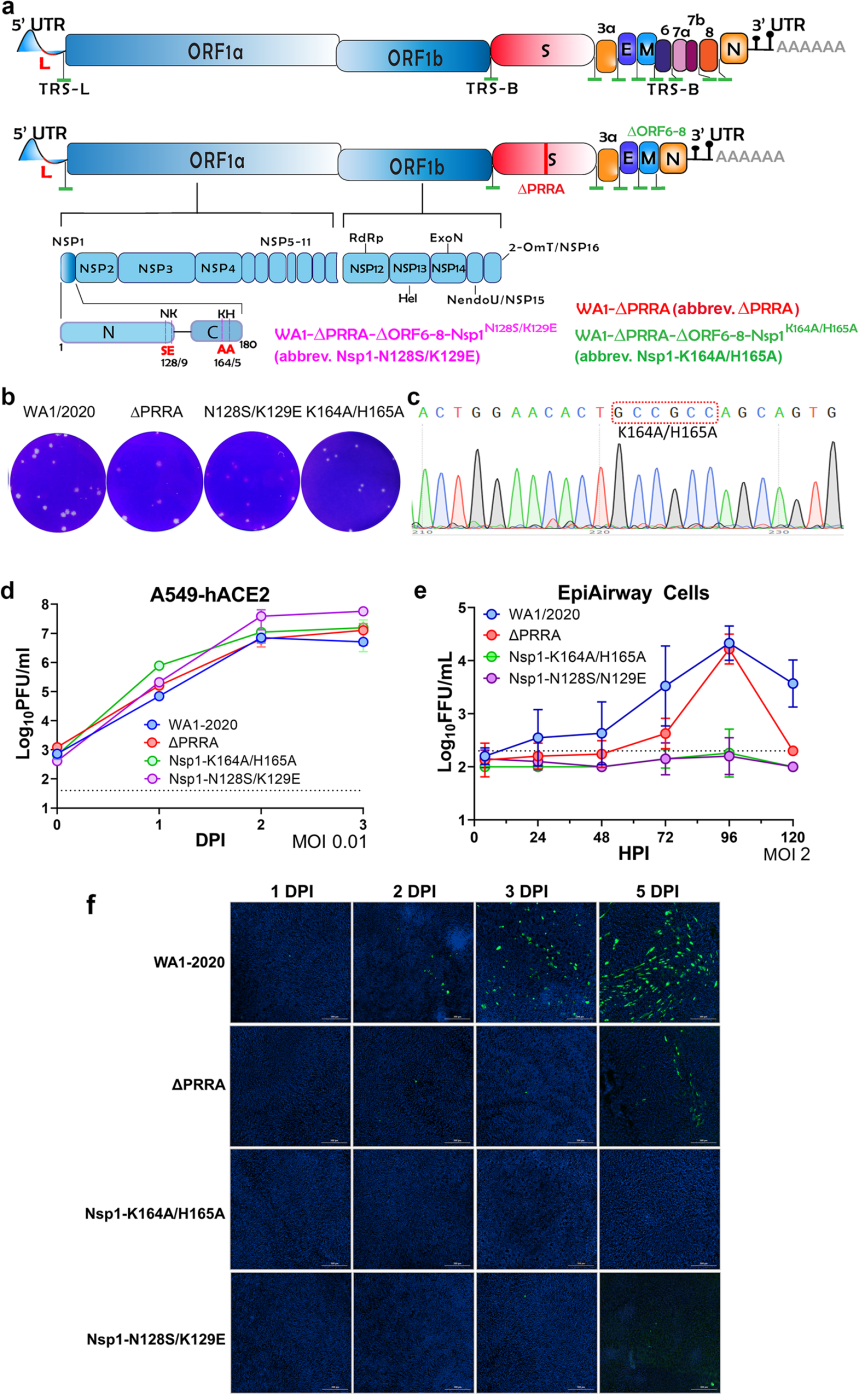
Rational attenuation of SARS-CoV-2 WA1/2020. **a** Top, genome organization of SARS-CoV-2. Leader sequence (red), transcriptional regulatory sequence within the leader sequence (TRS-L) and within the body (TRS-B) are highlighted in green. Bottom, either the polybasic insert “PRRA” (red) alone or together with ORF6-8 (green) were removed from the WA1/2020 genome. Locations of K164A/H165A and N128S/K129E are indicated at the bottom left of the figure. **b** Representative images of plaques formed by individual recombinant virus in Vero E6 cells. **c** Sanger sequencing result of Nsp1-K164A/H165A virus after passage 5. **d** A549-hACE2 cells were inoculated with indicated virus (*n* = 3 biological replicates) at multiplicity of infection (MOI) of 0.01. Output virus in the supernatants were determined on Vero E6 cells by plaque assay. **e** MatTek EpiAirway cells in 24-well plates were inoculated with indicated virus at MOI of 2. Supernatants collected at 2 (*n* = 6), 24 (*n* = 6), 48 (*n* = 5), 72 (*n* = 4), 96 (*n* = 3), and 120 (*n* = 2) hours post-infection (HPI) were titrated using a focus-forming assays biological replicates. Error bars for **d** and **e** indicate standard deviation. Data presented are representatives of two independent experiments with multiple biological replicates. Source data are provided as a Source data file. **f** MatTek EpiAirway cells from (**e**) were fixed in 10% formalin followed by immunostaining of nucleocapsid protein (in green) at 1, 2, 3, 5 dpi with one biological replicate per time point. Nuclei were counterstained by DAPI (in blue). Scale bar, 300 μm.

Although a single mutation may attenuate a virus, a LAV that differs from the wild-type virus by only one or two mutations poses an inherent safety concern due to the possibility of reversion. For that reason, we made the following modifications to the ancestral WA1/2020 viral genome: First, the polybasic insert (*PRRA*) immediately upstream of the furin cleavage site was removed from the virus. Such a modification abolishes the S1/S2 cleavage of the spike protein and significantly reduces infection of the lung^16,26^. Second, known IFN antagonists, ORFs6-8, were deleted from the viral genome. Thirdly, a pair of mutations (*K164A/H165A*) was introduced into the C-terminus of Nsp1 as we previously showed that these mutations significantly reduced cytotoxicity of SARS-CoV-2^27^. The three-pronged genetic modifications are to decrease infection of the lung, reduce inflammation and interferon antagonism, alleviate Nsp1-mediated toxicity. Ultimately, a recombinant virus termed “WA1-ΔPRRA-ΔORF6-8-Nsp1^K164A/H165A^” was obtained. For comparison purpose, we also generated two other recombinant viruses, WA1-ΔPRRA and WA1-ΔPRRA-ΔORF6-8-Nsp1^N128S/K129E^, respectively (Fig. 1a). WA1-ΔPRRA only has the polybasic insert removed, whereas WA1-ΔPRRA-ΔORF6-8-Nsp1^N128S/K129E^ has both the polybasic insert and ORFs6-8 deleted and then contains a pair of mutations (*N128S/K129E*) that did not efficiently abolish Nsp1-mediated cytotoxicity as did *K164A/H165A*^27^. All recombinant viruses formed plaques in Vero E6 cells and reached titers to 10^7^ pfu/ml (Fig. 1b). For brevity, the three recombinant viruses are also referenced as “ΔPRRA”, “Nsp1-K164A/H165A”, and “Nsp1-N128S/K129E” throughout the text. To monitor the genome stability, recombinant Nsp1-K164A/H165A virus was passaged five times in Vero E6 cells and human lung cell line H1299/hACE2 before viral genome sequencing. Passaging in H1299/hACE2 did not result in any new mutations, whereas passaging in Vero E6 cells led to a few single nucleotide variants (SNVs) outside the designed region of mutations (Supplementary Fig. 1). Sanger sequencing was also performed to detect the presence of *K164A/H165A* in Nsp1 after passage 5 (Fig. 1c). Overall, no reversion was found, and the genome of recombinant viruses appeared to be stable after cell passages in H1299/hACE2 cells. The three recombinant viruses grew with similar kinetics in A549-hACE2 cells to comparable titers (Fig. 1d). Compared to the ancestral virus (WA1/2020), all three recombinant viruses poorly infected primary human airway cells that were cultured at the air-liquid interface except that ΔPRRA mutant yielded infectious virus after 4 days. Nsp1-K164A/H165A and Nsp1-N128S/K129E displayed nearly no infectivity on primary cells over a period of 5 days (Fig. 1e, f).

### Attenuation of Nsp1-K164A/H165A in K18-hACE2 transgenic mice

**Fig. 2:**
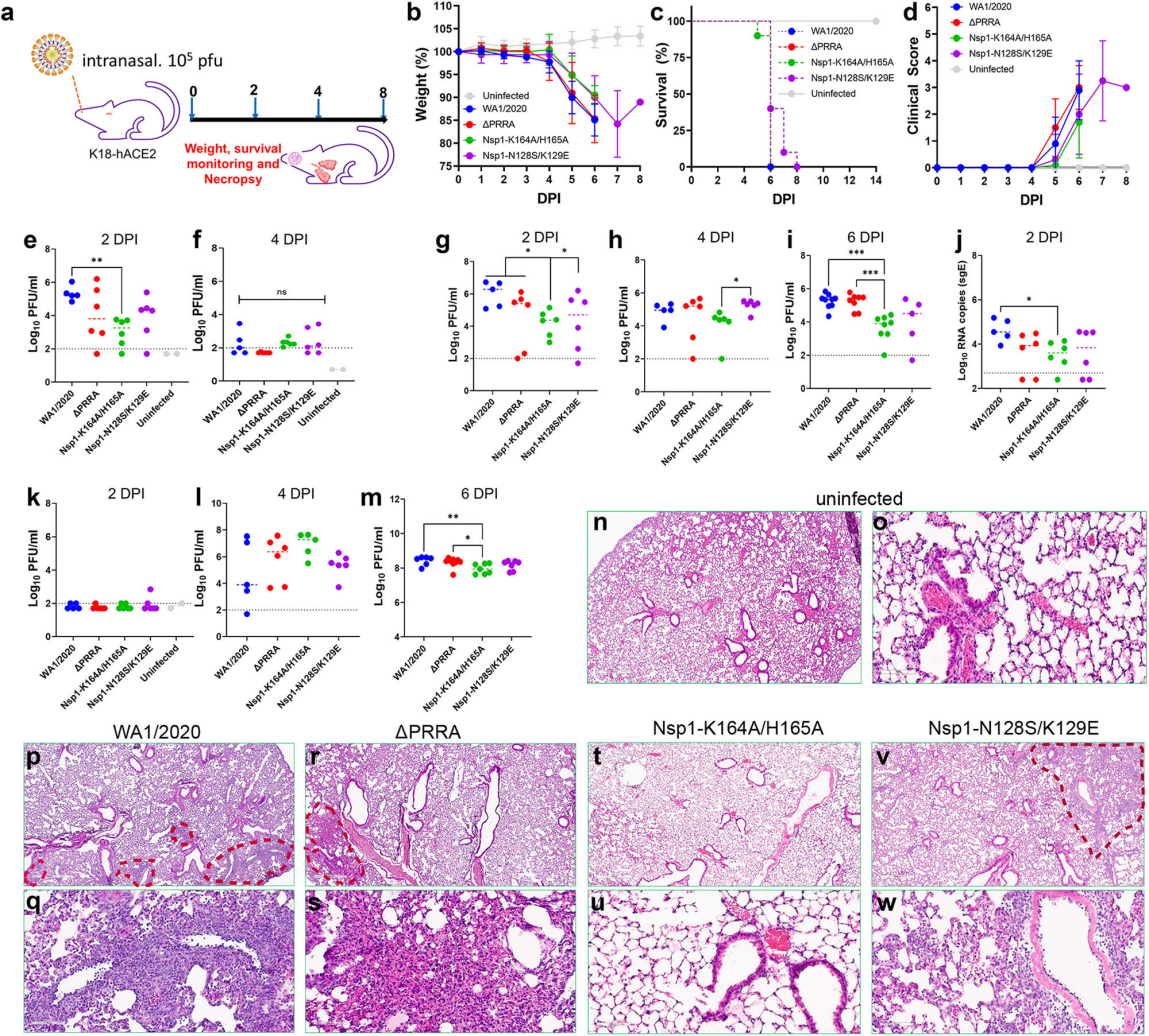
Attenuation of Nsp1-K164A/H165A in the respiratory tract of K18-hACE2 mice. **a** Overall design of the study. **b**–**d** Weight loss (**b**), survival (**c**), and clinical scores (**d**) were recorded in infected mice for up to 8 days following infection. Data in **b**–**d** reflect *n* = 10 mice/group from one experiment. **e**, **f** infectious viral titers of nasal turbinates at 2 dpi (**e**) or 4 dpi (**f**) were determined by plaque-forming assays. Each solid circle represents one animal. **p = 0.0229, one-way ANOVA. ns, non-significant. **g**–**i** infectious viral titers of lung homogenates at 2 dpi (**g**), 4 dpi (**h**), and 6 dpi (**i**). Median log_10_-transformed infectious titers at 2 dpi are 6.3 (IQR, 5.2 to 6.7), 5.4 (IQR, 2.2 to 5.8), 4.3 (IQR, 3.3 to 4.8), and 4.7 (IQR, 2.4 to 5.8) for WA1/2020, ΔPRRA, Nsp1-K164A/H165A, and Nsp1-N128S/K129E, respectively. Median log_10_-transformed infectious titers at 6 dpi are 5.4 (IQR, 5.0 to 5.5), 5.4 (IQR, 4.7 to 5.5), 3.9 (IQR, 3.3 to 4.2), and 4.5 (IQR, 2.5 to 5.2) for WA1/2020, ΔPRRA, Nsp1-K164A/H165A, and Nsp1-N128S/K129E, respectively. *p < 0.05, ***p < 0.001. **j** Log_10_-transformed sgRNA titers (E gene) were quantified by RT-qPCR. *p < 0.05. **k**–**m** Infectious viral titers of brain homogenates at 2 dpi (**k**), 4 dpi (**i**), and 6 dpi (**m**). *p < 0.05, **p < 0.01. Data in **e**–**m** reflect *n* = 5 WA1/2020, *n* = 6 ΔPRRA, *n* = 6 Nsp1-K164A/H165A, *n* = 6 Nsp1-N128S/K129E, or *n* = 2 uninfected mice/group from one experiment. Statistical analyses were done by one-way ANOVA in this figure. Error bars indicate standard deviation. **n**–**w** H&E stained lungs from uninfected (**n**, **o**), WA1/2020 (**p, q**), ΔPRRA (**r**, **s**), Nsp1-K164A/H165A (**t**, **u**), Nsp1-N128S/K129E (**v**, **w**). Red dotted lines denote areas of impact (consolidated). (**o**), (**q**), (**s**), (**u**), and (**w**) are closeup images of (**n**), (**p**), (**r**), (**t**), and (**v**), respectively. Experiments were conducted once, with multiple biological replicates. Source data are provided as a Source data file. Scale bar in (**n**), (**p**), (**r**), (**t**), and (**v**), 500 μm; in (**o**), (**q**), (**s**), (**u**), and (**w**), 90 μm.

To test attenuation in vivo, adult K18-hACE2 transgenic mice were divided into five groups (*n* = 10/group) and intranasally inoculated with 10^5^ plaque forming unit (PFU) of WA1/2020, ΔPRRA, Nsp1-K164A/H165A, and Nsp1-N128S/K129E or left uninoculated. Weight, survival, and clinical signs of illness were monitored for eight days. Notably, all infected mice succumbed to the infection by day 8 (Fig. 2a–d). Because encephalitis following SARS-CoV-2 infection is known to cause lethality in this model^28–34^, possible attenuation of the virus in the respiratory tract could have been masked by the lethality caused by encephalitis. To compensate for the limitations of the K18-hACE2 mouse model, we quantified the viral loads in nasal turbinates, lungs, and brains at 2, 4, 6 days post infection (dpi). In nasal turbinates, the median log_10_-transformed infectious titers peaked by 2 dpi at 5.3 [interquartile range (IQR), 5.0 to 5.7], 3.8 (IQR, 2.6 to 5.7), 3.2 (IQR, 2.2 to 3.7), and 4.3 (IQR, 2.8 to 4.7) for WA1/2020, ΔPRRA, Nsp1-K164A/H165A, and Nsp1-N128S/K129E infected mice, respectively. Infectious titers subsided to approximately the lower limit of quantification by 4 dpi (Fig. 2e, f). In the lungs of Nsp1-K164A/H165A infected mice, infectious viral titers were ∼2log_10_ lower compared to WA1/2020-infected animals at 2 and 6 dpi (Fig. 2g–j). The lung viral loads in Nsp1-K164A/H165A infected mice also trended lower than those from ΔPRRA and Nsp1-N128S/K129E groups, reaching statistical significance at 4 and 6 dpi. The viral loads in the brain, however, were largely comparable among the four infected groups except that at 6 dpi the Nsp1-K164A/H165A group had the lowest viral titers (Fig. 2k–m). Interestingly, at 2 dpi, there was very little detectable infectious virus in the brain, but the viral load in the brains rose in delayed kinetics as opposed to that in the respiratory tract. High viral loads in the brains of the Nsp1-K164A/H165A group at 6 dpi were coupled with a lack of pathology in lung tissues. Haematoxylin and eosin (H&E) staining (Fig. 2n–w) found lung lesions in WA1/2020, ΔPRRA, and Nsp1-N128S/K129E infected mice. Generally, there were peribronchiolar and perivascular immune infiltration. By contrast, Nsp1-K164A/H165A infected lungs had less than 1% impacted area with little pathology, which are nearly indistinguishable from the uninfected mice. Altogether, these results demonstrated that the Nsp1-K164A/H165A virus was primarily attenuated in the lower respiratory tract of K18-hACE2 mice but was still neuroinvasive in this highly sensitive mouse model.

### Attenuation of Nsp1-K164A/H165A in Syrian hamsters

**Fig. 3:**
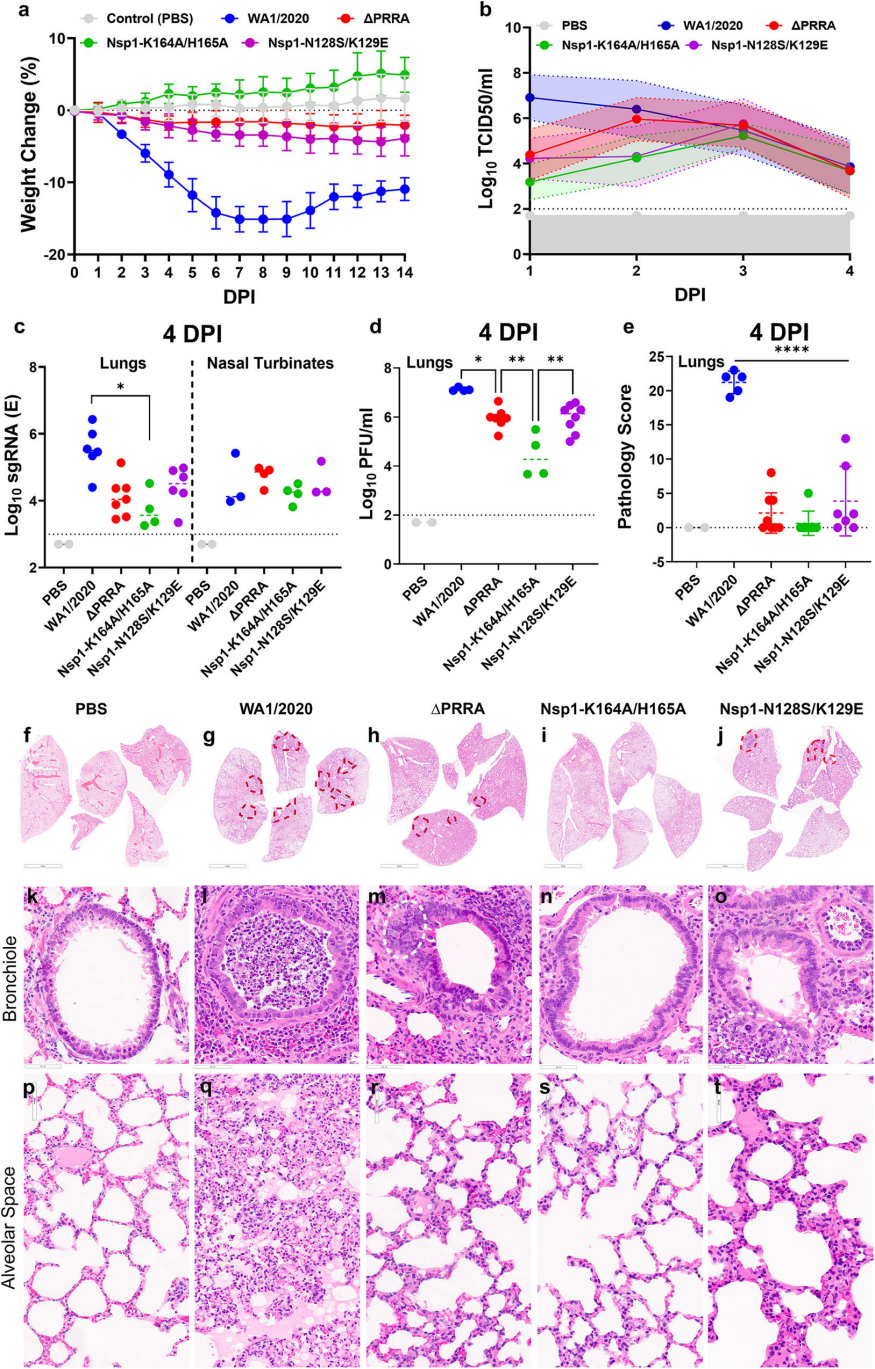
Attenuation of Nsp1-K164A/H165A in Syrian hamsters. **a** Weight loss of Syrian hamsters after infection with 10^4^ PFU of WA1/2020 (*n* = 7), ΔPRRA (*n* = 7), Nsp1-N128S/K129E (*n* = 7), Nsp1-K164A/H165A (*n* = 4), or PBS (*n* = 11). Error bars indicate standard deviation. **b** Median Log_10_ infectious titers in nasal wash samples WA1/2020 (5.9, IQR 4.3 to 6.8, *n* = 11), ΔPRRA (5.0, IQR 3.7 to 5.9, *n* = 15), Nsp1-N128S/K129E (4.3, IQR 3.8 to 5.4, *n* = 17), Nsp1-K164A/H165A (3.9, IQR 3.3 to 4.9, *n* = 8), or PBS (*n* = 2). Dotted, colored lines and color-filled areas marked by dotted lines indicate error bands (standard deviations). The limit of quantification is 200 TCID_50_/ml. Data for WA1/2020, ΔPRRA, Nsp1-N128S/K129E, and Nsp1-K164A/H165A reflect hamsters from two independent experiments. Error bars indicate standard deviation. **c** Log_10_-transformed sgRNA (E gene) titers in the lung for WA1/2020 (*n* = 6), ΔPRRA (*n* = 7), Nsp1-K164A/H165A (*n* = 4), Nsp1-N128S/K129E (*n* = 6), or PBS (*n* = 2) and nasal turbinates at 4 dpi for WA1/2020 (*n* = 3), ΔPRRA (*n* = 4), Nsp1-K164A/H165A (*n* = 4), Nsp1-N128S/K129E (*n* = 3), or PBS (*n* = 2). Each solid circle denotes one animal. *p = 0.0011, one-way ANOVA. **d** Log_10_-transformed infectious titers in the lung at 4 dpi for WA1/2020 (*n* = 4), ΔPRRA (*n* = 7), Nsp1-K164A/H165A (*n* = 4), Nsp1-N128S/K129E (*n* = 8), or PBS (*n* = 2). *p < 0.05, **p < 0.01, one-way ANOVA. **e** Cumulative histopathology scores of infected lungs at 4 dpi for WA1/2020 (*n* = 5), ΔPRRA (*n* = 8), Nsp1-K164A/H165A (*n* = 8), Nsp1-N128S/K129E (*n* = 7), or PBS (*n* = 2). ****p < 0.0001, one-way ANOVA. Source data are provided as a Source data file. **f**─**j** Representative images from H&E stained lungs from hamsters infected by PBS (**f**), WA1-2020 (**g**), ΔPRRA (**h**), Nsp1-K164A/H165A (**i**), Nsp1-N128S/K129E (**j**). Data are from *n* = 4 hamsters/group from one experiment. Red dotted lines denote areas of impact (consolidated). scale bar in **f**─**j**, 5 mm. **k**─**o** are representative images of bronchioles corresponding to **f**─**j**, respectively. **l** Massive luminal immune infiltrates. **m** Peribronchiolar infiltrates (white circle). **o** Peribronchiolar infiltrates (white circle). Scale bar in **k**─**o**, 60 μm. **p**─**t** are representative images of alveolar space corresponding to (**f**─**j**), respectively. **q** Oedema, immune infiltrates, alveolar wall thickening. **r** Alveolar wall thickening, loss of alveolar space, type II pneumocyte hyperplasia. **t** Loss of alveolar space and alveolar wall thickening. Scale bar in **p**─**t**, 60–80 μm.

Syrian hamsters are highly susceptible to SARS-CoV-2 and have been widely used in COVID-19 research^35–37^. Given the intrinsic shortcomings of the K18-hACE2 mouse model due to fatal neuroinvasion, we further evaluated the possible attenuation of Nsp1-K164A/H165A in hamsters. To this end, five groups of 6-month-old Syrian hamsters were intranasally inoculated with 10^4^ PFU of each virus or left uninoculated. This inoculum has consistently yielded weight loss, clinical signs, and lung pathology in Syrian hamsters^38^. Shown in Fig. 3a, WA1/2020-infected hamsters showed 18% weight loss on 7 dpi, whereas ΔPRRA, and Nsp1-N128S/K129E groups displayed no more than 5% weight loss over a period of 14 days. Nsp1-K164A/H165A infected animals (*n* = 8), like the uninfected group, had no weight loss at all through the study. During the first four days following infection, the log_10_-transformed infectious viral titers in nasal wash samples were measured by a TCID_50_ assay. Shown in Fig. 3b, the infectious titers from Nsp1-K164A/H165A infected animals were about two log_10_ lower than that of WA1/2020-infected hamsters at 1 and 2 dpi and trended lower than those from ΔPRRA and Nsp1-N128S/K129E-infected animals. Infectious nasal viral titers from all groups subsided to just above the limit of quantification at 4 dpi. Similarly, subgenomic RNA (sgRNA) titers in nasal turbinates from all groups were just above limit of quantification at 4 dpi (Fig. 3c). Log_10_-transformed sgRNA copies per ml in lung homogenates were 5.5 (IQR, 5.1 to 6.1), 4.0 (IQR 3.5 to 4.4), 3.5 (IQR 3.3 to 4.3), 4.5 (IQR 4.0 to 4.9), for WA1/2020, ΔPRRA, Nsp1-K164A/H165A, and Nsp1-N128S/K129E infected hamsters, respectively, at 4 dpi (Fig. 3c); median log_10_-transformed infectious viral titers in lung homogenates were 7.1 (IQR 7.0 to 7.2), 6.0 (5.8 to 6.1), 4.3 (IQR 3.7 to 5.3), 6.1 (IQR 5.4 to 6.4) for WA1/2020, ΔPRRA, Nsp1-K164A/H165A, and Nsp1-N128S/K129E infected hamsters, respectively (Fig. 3d). RNAscope revealed that viral RNA was present only along the bronchial epithelium in Nsp1-K164A/H165A-infected hamsters and the amount of staining was much less than the other three infected groups (Supplementary Fig. 2). Overall, viral loads of Nsp1-K164A/H165A-infected hamsters were 100–1000-fold lower than WA1/2020-infected animals and were half-log lower than ΔPRRA and Nsp1-N128S/K129E-infected animals. The lung pathology was subsequently scored. Once again, Nsp1-K164A/H165A-infected hamsters had minimal histopathological changes in the lung at 4 dpi (Fig. 3e). By contrast, WA1/2020 infection induced massive peribronchiolar edema and perivascular immune cell infiltrates, which led to significant consolidation (Fig. 3f–t). ΔPRRA and Nsp1-N128S/K129E groups occasionally had areas where type II hyperplasia and immune infiltration were found. Nsp1-K164A/H165A infected hamsters showed minimal pathological changes in the lung, sometimes indistinguishable from uninfected animals. The pathology of trachea followed the same trend observed in the lung with Nsp1-K164A/H165A infected animals showing only minimal submucosal lymphoplasmacytic infiltrates (Supplementary Fig. 3). None of the infections led to noticeable changes in heart and other critical organs as we have previously reported^38^.

**Fig. 4:**
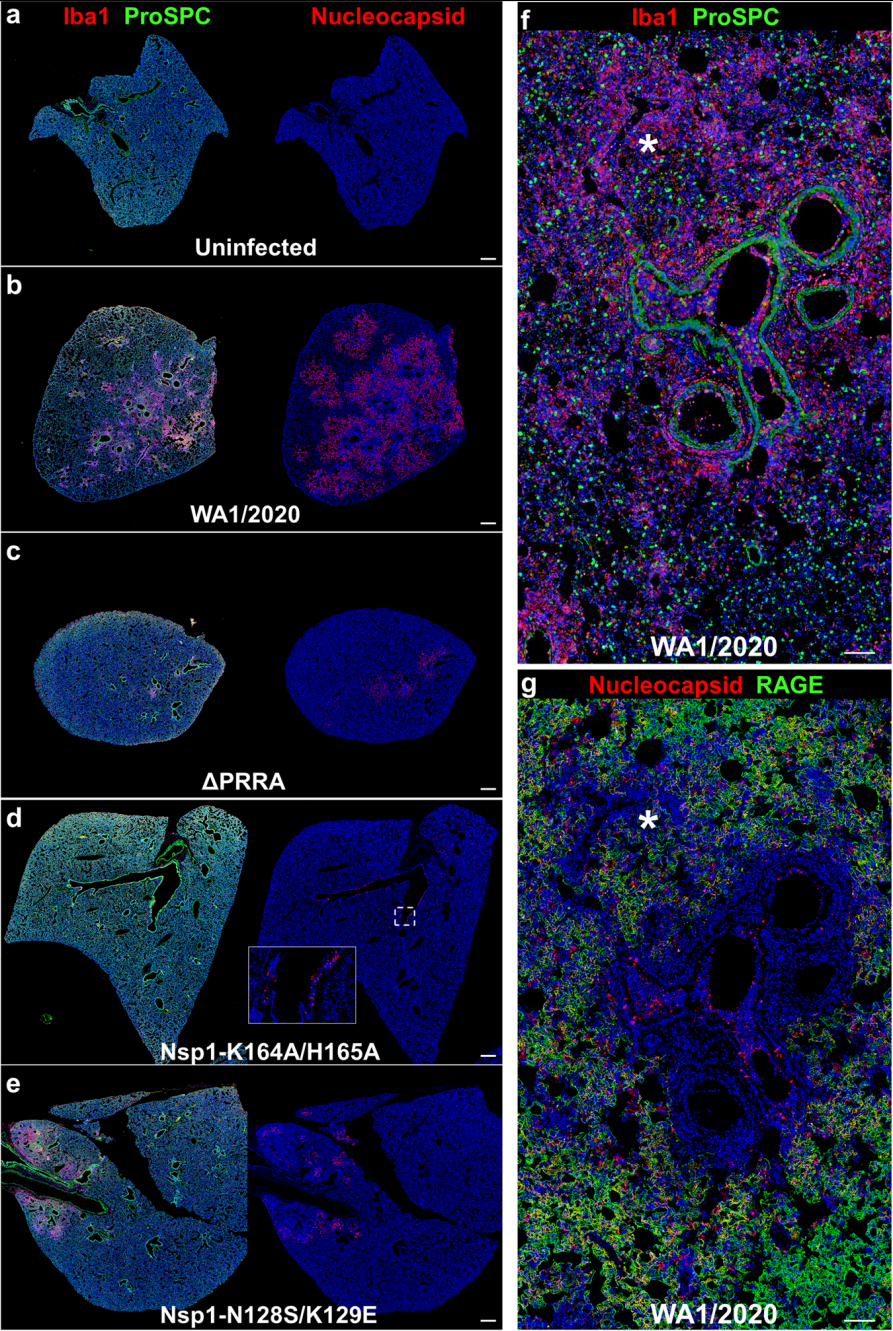
Attenuation of virus propagation, macrophage accumulation, and epithelial damage in Nsp1-K164A/H165A-infected hamster lungs. **a** Representative images of serial lung sections immunostained for Iba1 and prosurfactant protein C (ProSPC) or SARS-CoV-2 nucleocapsid protein after infection with PBS (**a**), WA1/2020 (**b**), ΔPRRA (**c**), Nsp1-K164A/H165A (**d**), Nsp1-N128S/K129E (**e**). Consolidated regions in WA1/2020-infected lungs with massive Iba1 positive macrophage infiltration around affected bronchioles (**b**). Alveolar epithelium surrounding consolidated regions in WA1/2020 stains prominently for viral nucleocapsid (**b**), while nucleocapsid staining is limited to bronchiolar epithelium in Nsp1-K164A/H165A (inset, **d**). **f** Digitally magnified images of macrophage-rich consolidation regions in WA1/2020-infected lungs show loss of ProSPC-stained alveolar type 2 cells. Viral antigen staining of infiltrates within and surrounding affected bronchioles with loss of RAGE-expressing type 1 epithelium in the same regions with consolidated macrophages (asterisk, **g**). Nuclei were counterstained with Hoechst 33342 dye (blue). Scale bars: 500 mm (**a**–**e**), 100 mm (**f**, **g**). Experiments were conducted once, with multiple biological replicates.

Immunofluorescence analyses showed that regions of consolidation in WA1/2020-infected lungs were dominated by Iba1-expressing macrophages (Fig. 4). Consolidated Iba1 staining was not detected or minimal in uninfected, ΔPRRA and Nsp1-K164A/H165A groups while consolidated Iba1 regions were visible in Nsp1-N128S/K129E-infected animals. Prominent staining for viral nucleocapsid was present in alveolar epithelium surrounding consolidated regions and within affected bronchioles in WA1/2020 (Fig. 4a, b). Nucleocapsid staining was limited to bronchiolar epithelium in Nsp1-K164A/H165A group (Fig. 4a), but reached alveolar epithelium in ΔPRRA and Nsp1-N128S/K129E infected animals. Reduced staining for RAGE and ProSPC, markers of type 1 and type 2 epithelial cells, respectively, highlighted the excessive epithelial damage in regions of consolidation (Fig. 4f, g).

**Fig. 5:**
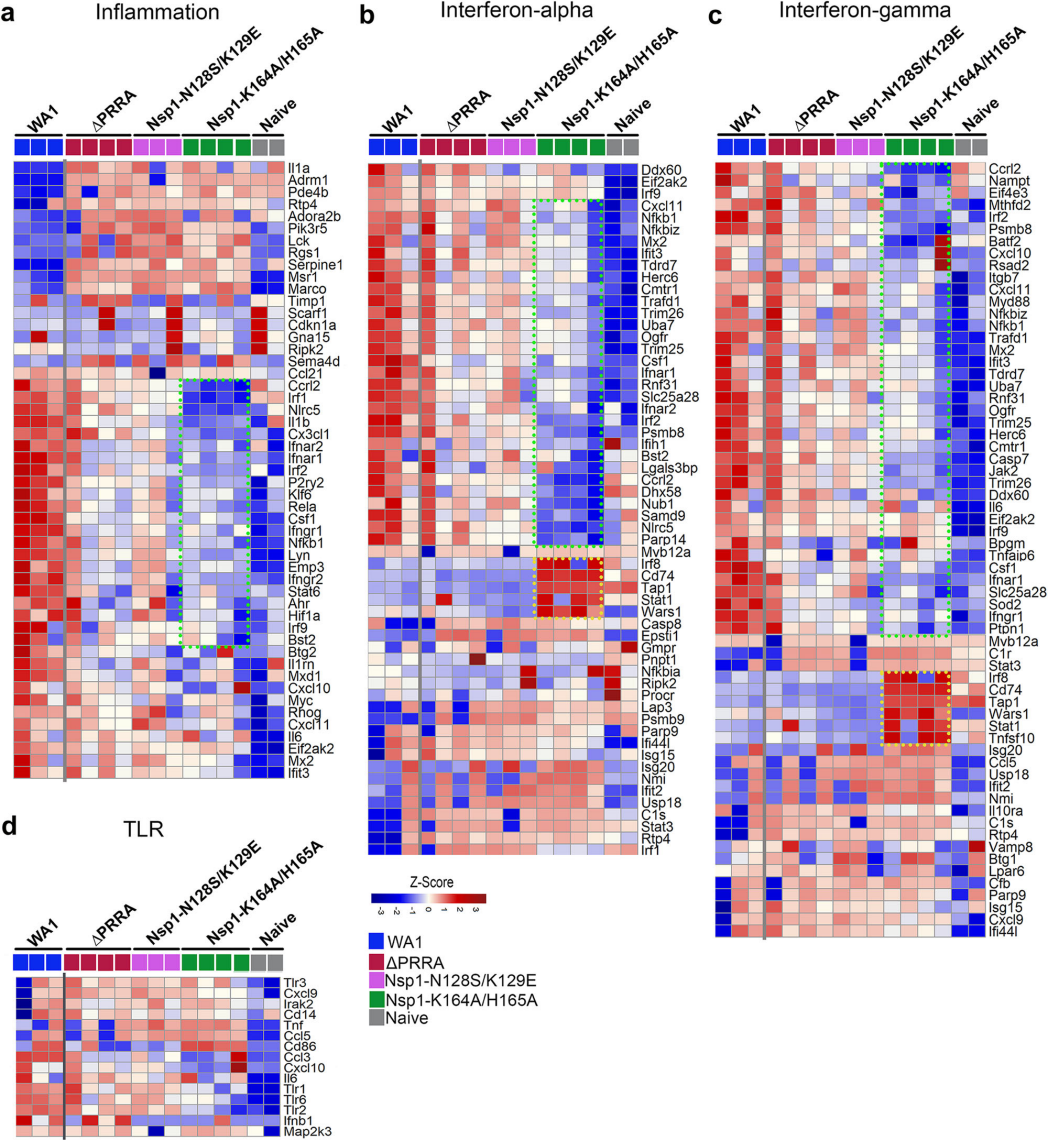
Heatmap analysis of interferon and inflammation signaling pathways in nasal turbinates. Genes associated with inflammation (**a**), interferon-alpha response (**b**), interferon-gamma response (**c**), and TLR responses (**d**) are presented in this figure. Each solid square represents one hamster. Five groups (uninfected, WA1/2020, ΔPRRA, Nsp1-K164A/H165A, and Nsp1-N128S/K129E) were coded in light charcoal, blue, red, green, and purple, respectively. The data represents the Z-scores derived from FPKM values of RNAseq transcriptomic analysis. Red, positive Z-Score denotes upregulation and blue, negative Z-score for downregulation. Genes in the yellow box were those that are specifically upregulated in Nsp1-K164A/H165A-infected hamsters. Genes in the green box were those that were expressed to a lesser degree in Nsp1-K164A/H165A group than in WA1/2020 or ΔPRRA or Nsp1-N128S/K129E groups.

To further assess changes at the molecular level, we isolated RNA from both nasal turbinates and lung homogenates at 4 dpi and performed RNAseq analyses. In nasal turbinates, comparison between WA1/2020 and ΔPRRA, Nsp1-N128S/K129E and Nsp1-K164A/H165A infected animals showed that WA1/2020 upregulated 34 and downregulated 17 genes in pathways of inflammation, upregulated 33 and downregulated 25 genes of pathways of type I IFN responses, and then upregulated 39 and downregulated 8 genes in pathways of type II IFN responses (Fig. 5). Most noticeably, Nsp1-K164A/H165A infection had least effects on the expressions of proinflammatory markers, such as *Mx2*, *Ifit3*, *Tlr6*, *Cxcl10*, and *Nfkb1* (Fig. 5a). Nsp1-K164A/H165A infection upregulated least numbers of genes of the interferon-alpha and gamma responses (Fig. 5b, c), presumably due to the lowest viral load among all tested groups. In some cases, gene expression profiles of Nsp1-K164A/H165A-infected hamsters were indistinguishable from those of uninfected hamsters. Strikingly, Nsp1-K164A/H165A specifically upregulated genes like *Irf8*, *Tap1*, and *Stat1*, all of which are important for antiviral defense (Fig. 5b). In the lungs, WA1-2020 induced 50 proinflammatory genes, 14 TLR signaling genes, 25 genes of the type I IFN and 37 genes of type II IFN pathways to higher levels than in ΔPRRA, Nsp1-K164A/H165A, and Nsp1-N128S/K129E infected hamsters. For those genes that were significantly downregulated in WA1/2020-infected animals, their expressions were restored in ΔPRRA, Nsp1-K164A/H165A, and Nsp1-N128S/K129E infected hamsters (Supplementary Fig. 4). Notably, we did not observe significant differences in terms of gene expression profiles in the lungs between ΔPRRA, Nsp1-K164A/H165A, and Nsp1-N128S/K129E infected animals, which may be a result of limited numbers of samples or an overall very low viral loads in the lungs.

### Intranasal immunization of Syrian hamsters with Nsp1-K164A/H165A induced potent humoral response and protected against WA1/2020 challenge

**Fig. 6:**
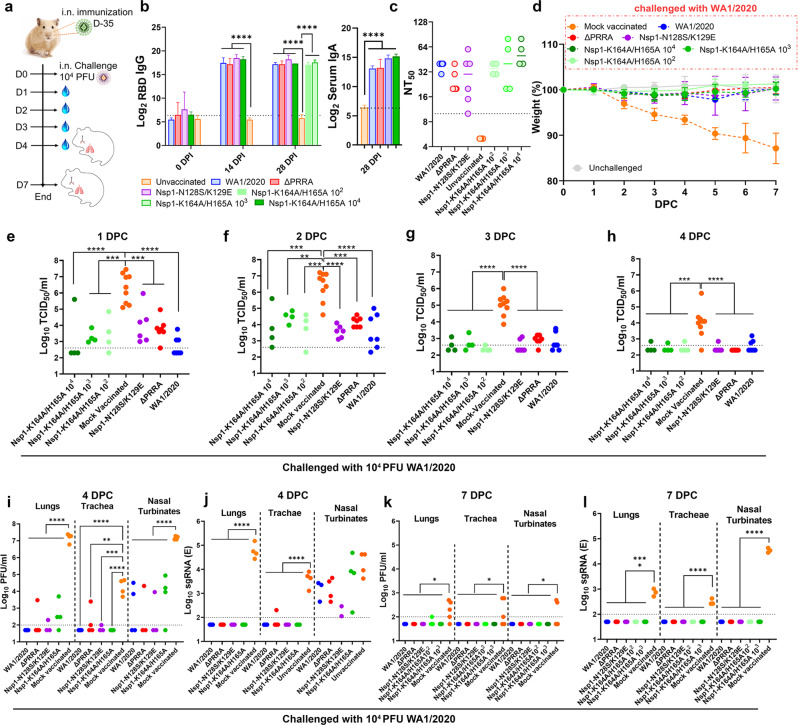
Intranasal immunization of Syrian hamsters with Nsp1-K164A/H165A induced potent humoral responses and protected against WA1/2020 challenge. **a** Overall study design. **b**, **c** Serum IgG antibody titers at 0 (*n* = 7), 14 (*n* = 7–11), 28 (*n* = 4–7) days and IgA antibody titer at 28 (*n* = 4–9) days post-immunization measured by RBD (spike) binding ELISA (**b**) or anti-spike neutralizing antibody titers at 28 dpi (**c**). ****p < 0.0001, one-way ANOVA. **d** Weight loss profiles of immunized and convalescent hamsters after re-challenge with WA1/2020. Error bars in **b** and **d** indicate standard deviation. **e**─**h** Infectious viral titers detected in nasal wash samples collected at 1, 2, 3, and 4 dpc. Median Log_10_ infectious titers at 1 dpc are 2.3 (IQR 2.3 to 3.1) for WA1/2020 convalescent hamsters, 3.8 (IQR 3.6 to 4.0) for ΔPRRA convalescent group, 3.8 (IQR 3.1 to 4.8) for Nsp1-N128S/K129E convalescent group, 2.3 (IQR 2.3 to 4.8) for Nsp1-K164A/H165A 10^4^ PFU vaccinated group, 3.2 (IQR 3.0 to 3.7) for 10^3^ PFU vaccinated group, 3.3 (IQR 2.5 to 4.5) for Nsp1-K164A/H165A 100 PFU vaccinated group, and 6.4 (IQR 5.3 to 7.1) for mock vaccinated hamsters. For **e** through **l**, **p < 0.01, ***p < 0.001, and ****p < 0.0001 based on one-way ANOVA. Sample sizes for **b**–**h**: for WA1/2020 (*n* = 7), ΔPRRA (*n* = 7), Nsp1-K164A/H165A (*n* = 4 per dose), Nsp1-N128S/K129E (*n* = 6), or unvaccinated (*n* = 9). **i**─**j** Infectious viral titers and sgRNA titers from tissues at 4 dpc, respectively. **k**, **l** infectious viral titers and sgRNA titers from tissues at 7 dpc, respectively. *P*-values are summarized based on the scheme: *p < 0.05, **p < 0.01, ***p < 0.001, ****p < 0.0001 using one-way ANOVA. Data shown in **b**–**h** reflect hamsters pooled from two independent experiments. Data shown in **i**–**l** reflect hamsters from one experiment. Each solid circle represents one animal. Source data are provided as a source data file.

**Fig. 7:**
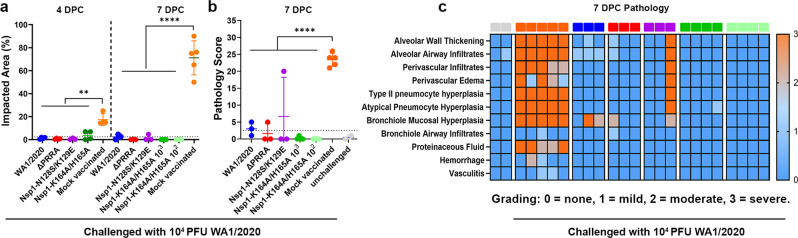
Syrian hamsters immunized with Nsp1-K164A/H165A displayed minimal lung pathology upon WA1/2020 challenge. This fugure summarizes the results after histopathological examination of the hamsters from Fig. 6i–l. **a** Percentages of impacted areas in the lungs at 4 and 7 dpc. **b** Cumulative histopathology scores in the lungs at 7 dpc. Error bars in **a** and **b** indicate standard deviation. *P*-values are summarized for **a** and **b** based on one-way ANOVA (**p < 0.01, ****p < 0.0001). **c** Heatmap of histopathology scores in the lungs at 7 dpc based on each category (see “Methods” for scoring criteria). Each solid square on top of the heatmap represents one hamster. Seven groups (uninfected, mock vaccinated and then challenged, WA1/2020 infected and then challenged, ΔPRRA infected and then challenged, Nsp1-N128S/K129E infected and challenged, immunized with 1000 PFU Nsp1-K164A/H165A and challenged, immunized with 100 PFU Nsp1-K164A/H165A and challenged) were coded in light gray, orange, blue, red, purple, green, and light green, respectively. Each solid shape represents one animal. Samples collected at 4 dpc for WA1/2020 (*n* = 4), ΔPRRA (*n* = 4), Nsp1-K164A/H165A (*n* = 4), Nsp1-N128S/K129E (*n* = 3), or mock vaccinated (*n* = 2) are combined from 2 separate experiments. Samples collected at 7 dpc for WA1/2020 (*n* = 3), ΔPRRA (*n* = 3), Nsp1-K164A/H165A (*n* = 4 for both doses), Nsp1-N128S/K129E (*n* = 3), mock vaccinated (*n* = 5), or unchallenged (*n* = 2) are also pooled from 2 separate experiments.

Based on the data obtained from above studies, Nsp1-K164A/H165A appears to be the most attenuated recombinant virus and hence was chosen for subsequent evaluation of immunogenicity and efficacy as a LAV candidate. To this end, adult Syrian hamsters were intranasally immunized with 10^2^, 10^3^, and 10^4^ PFU Nsp1-K164A/H165A. For comparison, we also inoculated hamsters with 10^4^ PFU WA1/2020, ΔPRRA, and Nsp1-N128S/K129E and housed animals until convalescence (Fig. 6a). A single dose of Nsp1-K164A/H165A induced binding and neutralizing antibodies (Fig. 6b, c) to levels that are comparable to those from WA1/2020-infected hamsters at 14 or 28 days after immunization. Interestingly, a dose of 100 PFU was just as potent as a dose of 10^4^ PFU regarding induction of antibodies. Immunized and convalescent hamsters were subsequently challenged with 10^4^ PFU WA1/2020 virus and monitored for 7 days before necropsy. Mock vaccinated hamsters lost more than 15% body weight by day 7 post-challenge (dpc), whereas immunized hamsters from all three dosage groups did not lose any weight (Fig. 6d). At 1- and 2-days post-challenge, infectious viral titers in nasal wash samples collected from immunized animals were 3 to 4 log_10_ lower than those from unvaccinated but challenged animals (Fig. 6e, f) and largely resolved by 4 dpc (Fig. 6g, h). Infectious viral titers and sgRNA titers in trachea and lungs were frequently below limit of quantification in many of the immunized and convalescent hamsters at 4 and 7 dpc (Fig. 6i–l). Viral loads in the nasal turbinates of the immunized and convalescent hamsters were at least 4 logs lower at 4 dpc and then went undetectable at 7 dpc. Lastly, single-dose immunization with Nsp1-K164A/H165A completely protected hamsters from developing pneumonia upon challenge, with nearly 0% consolidation and no histopathological changes at 4 and 7 dpc (Fig. 7a–c and Supplementary Fig. 5). Interestingly, we did not observe anamnestic antibody response in the Nsp1-K164A/H165A-vaccinated hamsters (Supplementary Fig. 6), likely reflecting the robust protection and minimal viral replication in these animals, as we have found in convalescent animals^38^.

## Discussion

For a pandemic respiratory pathogen like SARS-CoV-2, an important factor to consider when evaluating a LAV is safety, i.e., how pathogenic the vaccine virus is. Naturally, a poorly replicating virus is likely to be less immunogenic. We presented a rational approach to attenuate SARS-CoV-2 and abolish its pathogenicity while preserving immunogenicity. In K18-hACE2 transgenic mice and Syrian hamsters, viral loads of WA1-ΔPRRA-ΔORF6-8-Nsp1^K164A/H165A^ infected animals were 100−1000-fold lower in the nose and the lungs than those of WA1/2020-infected animals and were also noticeably lower than two comparators, WA1-ΔPRRA and WA1-ΔPRRA-ΔORF6-8-Nsp1^N128S/K129E^-infected animals. The attenuation of WA1-ΔPRRA-ΔORF6-8-Nsp1^K164A/H165A^ pathogenesis was further confirmed by the absence of lung pathology in infected animals. The mechanism of attenuation is likely threefold: (1) removal of the polybasic insert (*PRRA*) rendered the virus less infectious in the lung^26,39–44^; (2) removal of *ORF6-8* and the introduction of *Nsp1*^*K164A/H165A*^ further weakened the ability of the virus to antagonize IFNs; (3) K164A/H165A mutations also alleviated Nsp1-mediated cytotoxicity as we have recently reported^27^. In consistence, WA1-ΔPRRA-ΔORF6-8-Nsp1^K164A/H165A^-infected hamsters showed more pronounced attenuation of proinflammatory genes in the nasal turbinates compared to WA1-ΔPRRA and WA1-ΔPRRA-ΔORF6-8-Nsp1^N128S/K129E^-infected animals. Infection with WA1-ΔPRRA-ΔORF6-8-Nsp1^K164A/H165A^ was limited to bronchiolar epithelium and hence recruited nearly no macrophages into the lung. As we did not create a recombinant virus that only lacks ORFs6-8, we are unable to quantify the contribution of each modification to the observed attenuation in vivo. Nonetheless, WA1-ΔPRRA-ORF6-8-Nsp1^K164A/H165A^ appears to be the least pathogenic among the three recombinant viruses in animal models in terms of overall tissue viral load and pathology. Splitting attenuating modifications in three regions within the viral genome, rather than concentrating them, is likely to reduce the chances of reversion to virulence through a single recombinational event. Therefore, WA1-ΔPRRA-ORF6-8-Nsp1^K164A/H165A^ may be a safer candidate for a LAV or a challenge virus in human challenge studies than those attenuated SARS-CoV-2 with only one or two-mutation difference from the wild type.

Unlike vaccines that express only the spike protein as immunogen, LAVs confer broader and/or more durable protection because the whole organism is recognized by the host immune system. As to the humoral immune response, profiling antibody binding in 40 COVID-19 convalescent patients identified B cell epitopes derived from many viral proteins, including S, M, N, ORF1ab, ORF3a, ORF6, and ORF8^45^. In this study, as low as an inoculum of 100 PFU WA1-ΔPRRA-ΔORF6-8-Nsp1^K164A/H165A^ elicited potent humoral immune response and prevented hamsters from developing lung pathology. The observed protection was accompanied by more than 5-log_10_ reductions in viral loads in the lung and trachea following challenge. Impressively, immunized animals also displayed over 4-log_10_ reductions in nasal viral load. It is known that natural infection by SARS-CoV-2 induces both mucosal antibody responses and systemic antibody responses^46^. Secretory immunoglobulin A (IgA) is thought to play a major role in protecting the upper and lower respiratory tract from acute infection. Unfortunately, vaccines that are administered intramuscularly or intradermally potently induce IgG but not much secretory IgA^12,47^. By contrast, intranasal administration of a vaccine likely induces more potent mucosal immunity^48–50^. Ongoing efforts in our group are the development of appropriate reagents and assays to reliably determine mucosal antibody titers in hamsters and to evaluate cellular immunity induced by WA1-ΔPRRA-ΔORF6-8-Nsp1^K164A/H165A^. Future studies are also warranted to directly assess the effectiveness of intranasal delivery of WA1-ΔPRRA-ΔORF6-8-Nsp1^K164A/H165A^ against SARS-CoV-2 transmission in comparison to mRNA or protein-based vaccines.

We expect that administration of WA1-ΔPRRA-ΔORF6-8-Nsp1^K164A/H165A^ will activate broader cellular immunity that is cross-protective against variants of concern than spike-based vaccines^51,52^ because replication of WA1-ΔPRRA-ΔORF6-8-Nsp1^K164A/H165A^ offers many more targets to derive T cell epitopes. While this area of research is currently under investigation, if needed, variant-specific LAVs can be rapidly generated using our attenuation strategy. The furin cleavage site, ORFs 6–8, and the Nsp1-K164/H165 residues are conserved among all major SARS-CoV-2 variants of concern.

Although a single intranasal administration of WA1-ΔPRRA-ΔORF6-8-Nsp1^K164A/H165A^ protected against SARS-CoV-2-induced pneumonia in Syrian hamsters, we noted some limitations in the study. First, WA1-ΔPRRA-ΔORF6-8-Nsp1^K164A/H165A^ remains neurovirulent in K18-hACE2 mice at an inoculum of 10^5^ PFU. Whether this reflects an intrinsic neurovirulence of the virus or the limitation of the mouse model will need to be investigated. Second, we and others have shown that immunity acquired through natural infection does not lead to sterilizing immunity in nasal cavity^53–56^. Hence, even if immunization with WA1-ΔPRRA-ΔORF6-8-Nsp1^K164A/H165A^ significantly reduces SARS-CoV-2 infection and pathology, to achieve sterilizing immunity in the upper respiratory tract may not be a realistic goal. Finally, studies are needed to monitor immune responses over time to establish the durability of the protective response after intranasal vaccination with WA1-ΔPRRA-ΔORF6-8-Nsp1^K164A/H165A^.

In summary, our studies demonstrate the value of rationally attenuated SARS-CoV-2 in facilitating the development of new vaccines. Intranasal delivery of the attenuated SARS-CoV-2 induces potent humoral immunity, provides excellent protection, and possibly promotes sterilizing immunity in the lung. Our results support intranasal delivery of rationally attenuated SARS-CoV-2 as a promising platform for preventing COVID-19 and thus warrants further exploration.

## Methods

Research described here complies with all relevant ethical regulations and has been approved by the US Food and Drug Aministration Institutional Biosafety Committee. All critical reagents are listed in Supplementary Table 1.

### Cells and viruses

Vero E6 cell line (Cat # CRL-1586) was purchased from American Type Cell Collection (ATCC) and cultured in Eagle’s minimal essential medium (MEM) supplemented with 10% fetal bovine serum (Invitrogen) and 1% penicillin/streptomycin and L-glutamine. A549-hACE2 (NR-53821) cells were obtained from BEI Resources and maintained in DMEM supplemented with 5% penicillin and streptomycin, and 10% fetal bovine serum (FBS) at 37 °C with 5% CO_2_. Lenti-X cell line was purchased from Takarabio (Cat No. 632180) and maintained in DMEM supplemented with 5% penicillin and streptomycin, and 10% fetal bovine serum (FBS) at 37 °C with 5% CO_2_.

EpiAirway cells (AIR-100-HCF) and culturing media were purchased from MatTek. EpiAirway is a ready-to-use, 3D mucociliary tissue model consisting of normal, human-derived tracheal/bronchial epithelial cells cultured at the air-liquid interface (ALI). Cells were cultured in MatTek proprietary media for 2 days prior to usage. Mucus was washed off at the time of infection.

The SARS-CoV-2 isolate WA1/2020 (NR-52281, lot 70033175) was obtained from BEI Resources, NIAID, NIH, and had been passed three times on Vero cells and 1 time on Vero E6 cells prior to acquisition. It was further passed once on Vero E6 cells in our lab. The virus has been sequenced and verified to contain no mutation to its original seed virus.

### Production of SARS-CoV-2 recombinant virus

SARS-CoV-2 recombinant virus was generated using a 7-plasmid reverse genetic system which was based on the virus strain (2019-nCoV/USA_WA1/2020) isolated from the first reported SARS-CoV-2 case in the U.S.^57^. The initial 7 plasmids were generous gifts from Dr. P-Y Shi (UTMB). Upon receival, fragment 4 was subsequently subcloned into a low-copy plasmid pSMART LCAmp (Lucigen) to increase stability. To introduce *Nsp1*^*N128S/K129E*^ and *K164A/H165A* mutations, pUC57-CoV2-F1 plasmids containing mutated Nsp1 were first created by using overlap PCR method with the following primers:

M13F: gtaaaacgacggccagt

N128S/K129Ef: taagaacggtAGTGAGggagctggtggccatagtta

N128S/K129E r: caccagctccCTCACTaccgttcttacgaagaagaa

K164A/H165Af: aaaactggaacactGCcGCcagcagtggtgttacccgtga

K164A/H165Ar: gggtaacaccactgctgGCgGCagtgttccagttttcttgaa

NheIr: cacgagcagcctctgatgca

PCR fragments were digested by *Bgl* II/*Nhe* I and ligated into *Bgl* II/*Nhe* I digested F1 plasmid. The spike ΔPRRA mutation was introduced in to pUC57-CoV2-F6 by using overlap PCR with primers:

M13F: gtaaaacgacggccagt

ΔPRRA-f: actcagactaattctcgtagtgtagctagtcaatc

ΔPRRA-r: actagctacactacgagaattagtctgagtctgat

BglIIr: cagcatctgcaagtgtcact

PCR fragments were digested by *Kpn* I/*Bgl* II and ligated into *Kpn* I/*Bgl* II digested F6 plasmid.

To delete the ORF6-ORF8 region, an overlap PCR was performed using the following primers:

Mf: ttaattttagccatggcaga

ORF68f: tttgcttgtacagtaaacgaacaaactaaaatgtc

ORF68r: ttttagtttgttcgtttactgtacaagcaaagcaa

AvrIIr: gaagtccagcttctggccca

PCR fragments were digested by *Mlu* I/*Avr* II and ligated into *Mlu* I/*Avr* II digested pCC1-CoV-2-F7 plasmid. The resulted plasmids were validated by restriction enzyme digestion and Sanger sequencing.

In vitro transcription and electroporation were carried following procedures that were detailed elsewhere^58^. To recover the virus, the RNA transcript was electroporated into Vero E6 cells. Virus after passage 1 was titrated by plaque forming assay in Vero E6 cells and verified by deep sequencing.

### Hamster challenge experiments

Adult male outbred Syrian hamsters were previously purchased from Envigo and held at FDA vivarium. All experiments were performed within the biosafety level 3 (BSL-3) suite on the White Oak campus of the U.S. Food and Drug Administration. The animals were implanted subcutaneously with IPTT-300 transponders (BMDS), randomized, and housed 2 per cage in sealed, individually ventilated rat cages (Allentown). Hamsters were fed irradiated 5P76 (Lab Diet) ad lib, housed on autoclaved aspen chip bedding with reverse osmosis-treated water provided in bottles, and all animals were acclimatized at the BSL3 facility for 4–6 days or more prior to the experiments. The study protocol details were approved by the White Oak Consolidated Animal Care and Use Committee and carried out in accordance with the PHS Policy on Humane Care & Use of Laboratory Animals.

For challenge studies, adult (6–12 months old) Syrian hamsters were anesthetized with 3–5% isoflurane following procedures as described previously^38,59^. Intranasal inoculation was done by pipetting 10^4^ PFU or desirable doses of SARS-CoV-2 in 50 µl volume dropwise into the nostrils of the hamster under anesthesia. Following infection, hamsters were monitored daily for clinical signs and weight loss. Nasal wash samples taken on days 1-, 2-, 3-, and 4-day post infection to test for sgRNA by RT-qPCR and infectious virus by TCID50 in Vero E6 cells. Nasal washes were collected by pipetting ~160 µl sterile phosphate-buffered saline into one nostril when hamsters were anesthetized by 3–5% isoflurane. For tissue collection, a subset of hamsters was humanely euthanized by intraperitoneal injection of pentobarbital at 200 mg/kg and lungs for histopathology. Blood collection was performed under anesthesia (3–5% isoflurane) through gingival vein puncture or cardiac puncture when animals were euthanized.

### Mouse infection experiments

Female adult K18-hACE2 mice (12 weeks ago) were previously purchased from the Jackson laboratory and held at FDA vivarium. All experiments were performed within the biosafety level 3 (BSL-3) suite on the White Oak campus of the U.S. Food and Drug Administration. The study protocol details were approved by the White Oak Consolidated Animal Care and Use Committee and carried out in accordance with the PHS Policy on Humane Care & Use of Laboratory Animals.

For infection studies, mice were first anesthetized by 3–5% isoflurane. Intranasal inoculation was done by pipetting 10^5^ PFU SARS-CoV-2 in 50 µl volume dropwise into the nostrils of the mouse. Mice were weighed and observed daily. For tissue collections, mice were euthanized by CO_2_ overdose on days 2, 4, 6 as necessary.

### SARS-CoV-2 pseudovirus production and neutralization assay

Procedures as described previously^38,59^. In brief, 50 μL of SARS-CoV-2 S pseudovirions were pre-incubated with an equal volume of medium containing serum at varying dilutions at room temperature for 1 h, then virus-antibody mixtures were added to Vero E6 cells in a 96-well plate. After a 3 h incubation, the inoculum was replaced with fresh medium. Cells were lysed 48 h later, and luciferase activity was measured using luciferin-containing substrate. Controls included cell only control, virus without any antibody control, and positive control sera. The end-point titers were calculated as the last serum dilution resulting in at least 50% SARS-CoV-2 neutralization. A NIBSC anti-SARS-CoV-2 antibody (20/130) was included as a positive control.

### RNA isolation and qRT-PCR

Procedures as described previously^38,59^. In brief, RNA was extracted from 50 μl NW or 0.1 g tissue homogenates using QIAamp vRNA mini kit or the RNeasy 96 kit (QIAGEN) and eluted with 60 μl of water. 5 μL RNA was used for each reaction in real-time RT-PCR. When graphing the results in Prism 8, values below the limit of quantification (LoQ) were arbitrarily set to half of the LoQ values. Unless otherwise specified, the unit for RNA copies are as presented as Log_10_ RNA copies/5 μl nasal wash or Log_10_ RNA copes/0.1 g tissue homogenates,

### RNAseq

To prepare sequencing libraries, RNA was first extracted using the Trizol-chloroform method from the lung homogenates and nasal turbinates. The aqueous portion was further purified using RNeasy mini kit (Qiagen, Gaithersburg, MD). RNA quality was assessed using Agilent 2100 Bioanalyzer (Agilent Technologies, Santa Clara, CA), and the RNA integration numbers (RIN) were all greater than 9. An aliquot (1 μg) of each sample of total RNA was used to prepare sequencing libraries using Illumina Stranded Messenger RNA Prep (ligation based). The cDNA libraries were normalized and loaded onto a NovaSeq 6000 sequencer (Illumina, San Diego, CA) for deep sequencing of paired-end reads of 2 × 100 cycles. The sequencing reads for each sample were mapped to the respective reference genomes of *Mesocricetus auratus* (BCM_Maur_2.0) by Tophat (v2.1.2). Cufflinks (v2.2.1) was then used to assemble transcripts, estimate abundances and test for differential expression. The sequencing and initial data analysis using Qiagen CLC Genomics Workbench (version 21) was performed by FDA Next Generation Sequencing Core Facility. The sequencing and initial data analysis using Qiagen CLC Genomics Workbench (version 21) was performed by FDA Next Generation Sequencing Core Facility. Raw and processed data have been deposited to NCBI (GEO accession number GSE199922s).

Further data analysis was done using R Studio 1.4.1106 (http://www.R-project.org). Heatmaps were constructed using heatmap library. The gene list for signaling pathways was obtained from hallmark gene sets in Molecular Signatures Database (MSigDB)^60^. The figures were assembled in Adobe Photoshop. This work utilized the computational resources of the NIH HPC Biowulf cluster (http://hpc.nih.gov).

### Histopathology analyses

Procedures as described previously^38,59^. Tissues (hearts, brains, lungs, trachea, and nasal turbinates) were fixed in 10% neural buffered formalin overnight and then processed for paraffin embedding. The 5-μm sections were stained with hematoxylin and eosin for histopathological examinations. Images were scanned using an Aperio ImageScope. For hamster tissues, blinded samples were graded by a licensed pathologist for the following twelve categories: consolidation, alveolar wall thickening, alveolar airway infiltrates, perivascular infiltrates, perivascular edema, type II pneumocyte hyperplasia, atypical pneumocyte hyperplasia, bronchiole mucosal hyperplasia, bronchiole airway infiltrates, proteinaceous fluid, hemorrhage, vasculitis. Grading: 0 = none, 1 = mild, 2 = moderate, 3 = severe. A graph was prepared by summing up the score in each category. Mouse tissues were scored based on the following categories: consolidation, alveolar wall thickening, alveolar airway infiltrates, perivascular Infiltrates, perivascular edema, peribronchiolar infiltrates, type II pneumocyte hyperplasia, necrosis (alveoli and bronchiole), bronchiole mucosal hyperplasia, bronchiole airway infiltrates, proteinaceous fluid, hemorrhage, vasculitis. Grading scale: 0 = none, 1 = mild, 2 = moderate, 3 = severe.

### In situ hybridization (RNAscope)

To detect SARS-CoV-2 genomic RNA in FFPE tissues, ISH was performed using the RNAscope 2.5 HD RED kit, a single plex assay method (Advanced Cell Diagnostics; Catalog 322373) according to the manufacturer’s instructions. Briefly, Mm PPIB probe detecting peptidylprolyl isomerase B gene (housekeeping gene) (catalog 313911, positive-control RNA probe), dapB probe detecting dihydrodipicolinate reductase gene from Bacillus subtilis strain SMY (a soil bacterium) (catalog 310043, negative-control RNA probe) and V-nCoV2019-orf1ab (catalog 895661) targeting SARS-CoV-2 positive-sense (genomic) RNA. Tissue sections were deparaffinized with xylene, underwent a series of ethanol washes and peroxidase blocking, and were then heated in kit-provided antigen retrieval buffer and digested by kit-provided proteinase. Sections were exposed to ISH target probes and incubated at 40 °C in a hybridization oven for 2 h. After rinsing, ISH signal was amplified using kit-provided pre-amplifier and amplifier conjugated to alkaline phosphatase and incubated with a fast-red substrate solution for 10 min at room temperature. Sections were then stained with 50% hematoxylin solution followed by 0.02% ammonium water treatment, dried in a 60 °C dry oven, mounted, and stored at 4 °C until image analysis.

### Lung immunofluorescence analyses

Formalin-fixed paraffin-embedded (FFPE) lung sections 4 µm thick were dewaxed, rehydrated, and heat-treated in a microwave oven for 15 min in 10 mM Tris/1 mM EDTA buffer (pH 9.0). After cooling for 30 min at room temperature, heat-retrieved sections were blocked in PBST with 2.5% bovine serum albumin (BSA) for 30 min at RT followed by overnight incubation at 4 °C with primary antibodies in 1% BSA. Primary antibodies used included SARS nucleocapsid protein (1:800, Sino Biologicals, 40143-MM05), prosurfactant protein C (1:200, EMD Millipore, AB3786), Iba1 (1:100, Abcam, ab5076), and RAGE (1:400, Abcam, ab216329). Sections were rinsed and incubated with 1:500 secondary antibodies congujated with Alexa Fluor 488 (A-21206) and Alexa Fluor 647 (A-31571, A-21447) for 1 h at RT (ThermoFisher, Waltham, MA). Nuclei were counterstained with Hoechst 33342. For double labeling experiments, primary antibodies were mixed and incubated overnight at 4 °C. For negative controls, sections were incubated without the primary antibody or mouse and rabbit isotype antibody controls. Sections stained with conjugated secondary antibodies alone showed no specific staining. Whole-slide fluorescence imaging was performed using a Hamamatsu NanoZoomer 2.0-RS whole-slide digital scanner equipped with a ×20 objective and a fluorescence module #L11600. Analysis software NDP.view2 was used for image processing (Hamamatsu Photonics, Japan).

### TCID_50_

Procedures as described previously^38,59^. In brief, Vero E6 cells were plated the day before infection into 96-well plates at 1.5 × 10^4^ cells/well. On the day of the experiment, serial dilutions of 20 μl nasal wash samples were made in media and a total of six to eight wells were infected with each serial dilution of the virus. After 48 h incubation, cells were fixed in 4% PFA followed by staining with 0.1% crystal violet. The TCID_50_ was then calculated using the formula: log(TCID_50_) = log(do) + log(R) (f + 1). Where do represents the dilution giving a positive well, f is a number derived from the number of positive wells calculated by a moving average, and R is the dilution factor.

### Plaque assay

Nasal wash samples were 10-fold serially diluted and added to a 24-well plate containing freshly confluent with Vero E6 cells. For tissue samples, entire trachea or nasal turbinates or the left lobe of the lung (~0.2 g) were resuspended in 1 milliliter MEM and homogenized on a Precellys Evolution tissue homogenizer with a Cooling Unit (Bertin). Tissue homogenates were then 10-fold serially diluted and added to Vero E6. After 1 h the mixture was removed and replenished with Tragacanth gum overlay (final concentration 0.3%). Cells were incubated at 37 °C and 5% CO_2_ for 2 days, then fixed with 4% paraformaldehyde, followed by staining of cells with 0.1% crystal violet in 20% methanol for 5–10 min. The infectious titers were then calculated and plotted as plaque forming units per milliliter (PFU/ml).

### Focus-forming assay

ALI cell culture supernatants were 10-fold serially diluted in 96-well plates and dilutions added to 96-well black-well plates for fluorescent focus-forming assays in H1299-hACE2 cells. After 1 h the Tragacanth gum overlay (final concentration 0.3%) was added. Cells were incubated at 37 °C and 5% CO2 for 1 day, then fixed with 4% paraformaldehyde, followed by staining of cells with primary rabbit anti-nucleocapsid Wuhan-1 antibody (custom made by Genscript) overnight followed by secondary anti-rabbit Alexa-488 conjugated antibody and DAPI staining. The infectious titers were then counted using Gen5 software on a Cytation7 machine and calculated and plotted as focus-forming units per milliliter (FFU/ml).

### Measurement of antibody by ELISA

SARS-CoV-2 S and RBD antigens for ELISA were prepared in a baculovirus expression system using procedures as published elsewhere^61^. Briefly, Immunlon 2HB plates were coated with recombinant S or RBD protein at 1 µg/mL overnight at 4 °C. Test serum samples were pre-diluted in assay diluent (PBS containing 0.05% Tween-20 [PBST] and 10% fetal bovine serum), followed by serial two-fold dilutions of each sample in duplicates across the plate. Plates were incubated with the test serum samples for 2 h at 37 °C. After rigorous plate washes in a microplate washer, a secondary antibody (anti-hamster IgG) conjugated to HRP (6060-05, SouthernBiotech, Birmingham, AL) was added to wells at 1:4000 dilution (hamster IgG ELISA). Plates were incubated with secondary antibody for 1 h, washed, and ABTS/H_2_O_2_ peroxidase substrate (SeraCare, Gaithersburg, MD) was added to assay wells. After 20 to 30 min at ambient temperature, reactions were stopped with 1% SDS, and OD_405_ values were captured on the Versamax microplate reader with the Softmax Pro 7 software installed (Molecular Devices, San Jose, CA). The assay endpoint was a mean OD_405_ of 0.05 for duplicate wells for the full-length S ELISA and a mean OD_405_ of 0.01 for the RBD ELISA. The reciprocal of the highest serum dilution at which the mean OD_405_ value averaged ≥0.05 (full-length S ELISA) or ≥0.01 (RBD ELISA) was the IgG titer. To determine the serum IgA antibody titers, each test serum sample was diluted from 1:160. The sandwich Rabbit anti-hamster IgA antibody (sab 3001a, Brookwood Biomedical, Jemison, Alabama) was added at 1:4000 dilution followed by 1:4000 Goat anti-rabbit-HRP (4030-05, Southern Biotech, Birmingham, AL).

### Statistical analysis

One-way ANOVA was used to calculate statistical significance through GraphPad Prism (9.1.2) software for Windows, GraphPad Software, San Diego, California, USA.

### Reporting summary

Further information on research design is available in the Nature Portfolio Reporting Summary linked to this article.

## Supplementary information

Supplementary Information

Supplementary Data File

Reporting Summary

## Data Availability

The RNA-seq datasets have been submitted to The National Center for Biotechnology Information. Raw and processed data have been deposited to NCBI (GEO accession number GSE199922s). Metadata file submitted for GEO submission along with the fastq files are included in Supplementary Data File. All unique/stable reagents generated in this study are available from the Lead Contact with a completed Materials Transfer Agreement. Source data are provided with this paper.

## Source data

Source Data
