# Supplementary material for: Intranasal delivery of a rationally attenuated SARS-CoV-2 is immunogenic and protective in Syrian hamsters
Source: Nat Commun. 2022 Nov 10;13:6792. doi: 10.1038/s41467-022-34571-4 (PMC9648440; doi:10.1038/s41467-022-34571-4)
Supplement: Supplementary file 1 — Supplementary Information [file 41467_2022_34571_MOESM1_ESM.pdf]

Supplementary Materials for

Intranasal Delivery of a Rationally Attenuated SARS-CoV-2 Is Safe, Immunogenic and Protective in Syrian Hamsters

Shufeng Liu[1, †], Charles B. Stauft[1, †], Prabhuanand Selvaraj[1, †], Prabha Chandrasekaran[2, †], Felice D′Agnillo[3, †], Chao-Kai Chou[4], Wells W. Wu[4], Christopher Z. Lien[1], Clement A. Meseda[1], Cyntia L. Pedro[1], Matthew F. Starost[5], Jerry P. Weir[1], Tony T. Wang[1]*

[1]Division of Viral Products, Center for Biologics Evaluation and Research, Food and Drug Administration; Silver Spring, Maryland, USA.

[2]Laboratory of Clinical Investigation, National Institutes of Aging, National Institutes of Health, Baltimore, USA.

[3]Facility for Biotechnology Resources, Center for Biologics Evaluation and Research, U.S. Food and Drug Administration, Silver Spring, Maryland, USA.

[4]Division of Veterinary Resources, Diagnostic and Research Services Branch, National Institutes of Health, Rockville Pike, USA.

[5]These authors contributed equally.

*Correspondence to: Tony.Wang@fda.hhs.gov

The PDF file includes:

Supplementary Figs. 1-6

Supplementary Table 1

Other Supplementary Material for this manuscript includes the following:

Metadata of RNAseq Supplementary Data File

Supplementary Figures and Legends

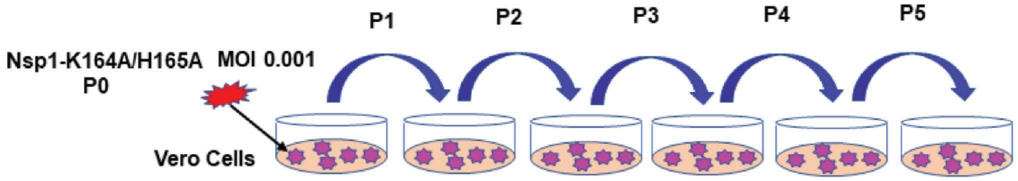

Region	Type	Reference	Allele	Frequency of SNV				
				P1	P2	P3	P4	P5
616	SNV	T	G			20.25		
616	SNV	T	T			79.41		
1393	SNV	A	T			22.64	21.83	
1393	SNV	A	A			77.25	78.17	
7486	SNV	A	T	100.00	100.00	100.00	100.00	99.62
7489	SNV	T	A	100.00	99.59	99.43	100.00	100.00
10897	SNV	A	C			23.65	24.83	
10897	SNV	A	A			76.19	74.83	
12520	SNV	T	C			29.89	33.02	
12520	SNV	T	T			70.11	66.98	
18060	SNV	T	C	99.53	98.87	99.19	99.79	98.81
21784	SNV	T	A			23.26	22.27	
21784	SNV	T	T			74.55	76.92	
22296	SNV	A	G			29.39	47.68	59.96
22296	SNV	A	A			70.61	52.32	40.04

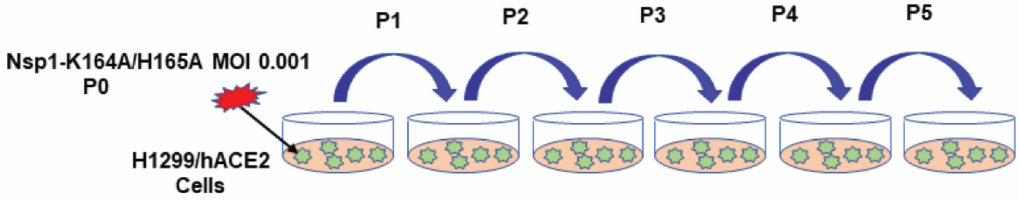

Region	Type	Reference	Allele	Frequency of SNV			
				P2	P3	P4	P5
7486	SNV	A	T	100.00	100.00	99.77	100.00
7489	SNV	T	A	99.75	99.82	100.00	100.00
18060	SNV	T	C	99.31	98.78	98.93	98.88

Supplementary Fig. 1: Summary of single nucleotide variants of Nsp1-K164A/H165A virus after cell passages. Passage 0 Nsp1-K164A/H165A virus (generated after electroporation of RNA into Vero E6 cells) was added to either Vero E6 cells or H1299/hACE2 cells at MOI of 0.001. When cytopathic effect was observed, supernatants were collected and titered by plaque assay and subsequently added at MOI of 0.001 to fresh cells for next round of passage. Virus collected after each passage was subject to RNA isolation. Library was prepared using Illumina total RNA ligation with ribozero protocol to remove ribosomal RNA. Sequencing was performed on a MiSeq instrument and 70 to 90% of reads were mapped

to WA1-ΔPRRA-ΔORF6-8-Nsp1$^{K164A/H165A}$. For variant calling, greater than 20% of frequency was used here. Notably, three silent mutations (in green) at nucleotide positions 7,486 (A-to-T change), 7,489 (T-to-A change), and 18,060 (T-to-C change) were inherited from the initial reverse genetics plasmids to differentiate the infectious clone-derived virus from the natural clinical isolate 2019-nCoV/USA_WA1/2020. These three mutations are not SNVs derived from cell passages.

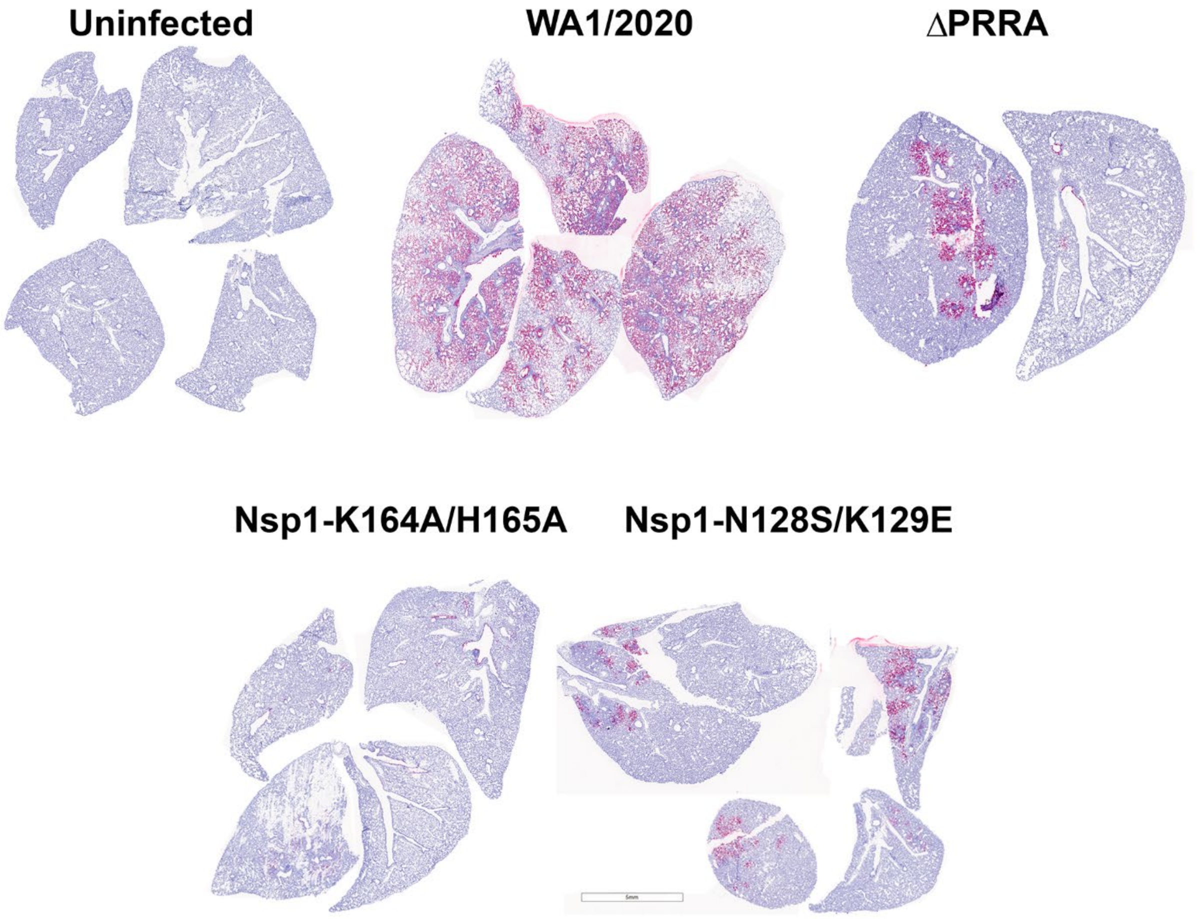

Supplementary Fig. 2: Presence of viral RNA in infected hamster lungs at 4 dpi. Viral RNA was detected using a specific probe (in red). Nuclei were stained by haematoxylin (blue). Experiments were conducted once, with multiple biological replicates.

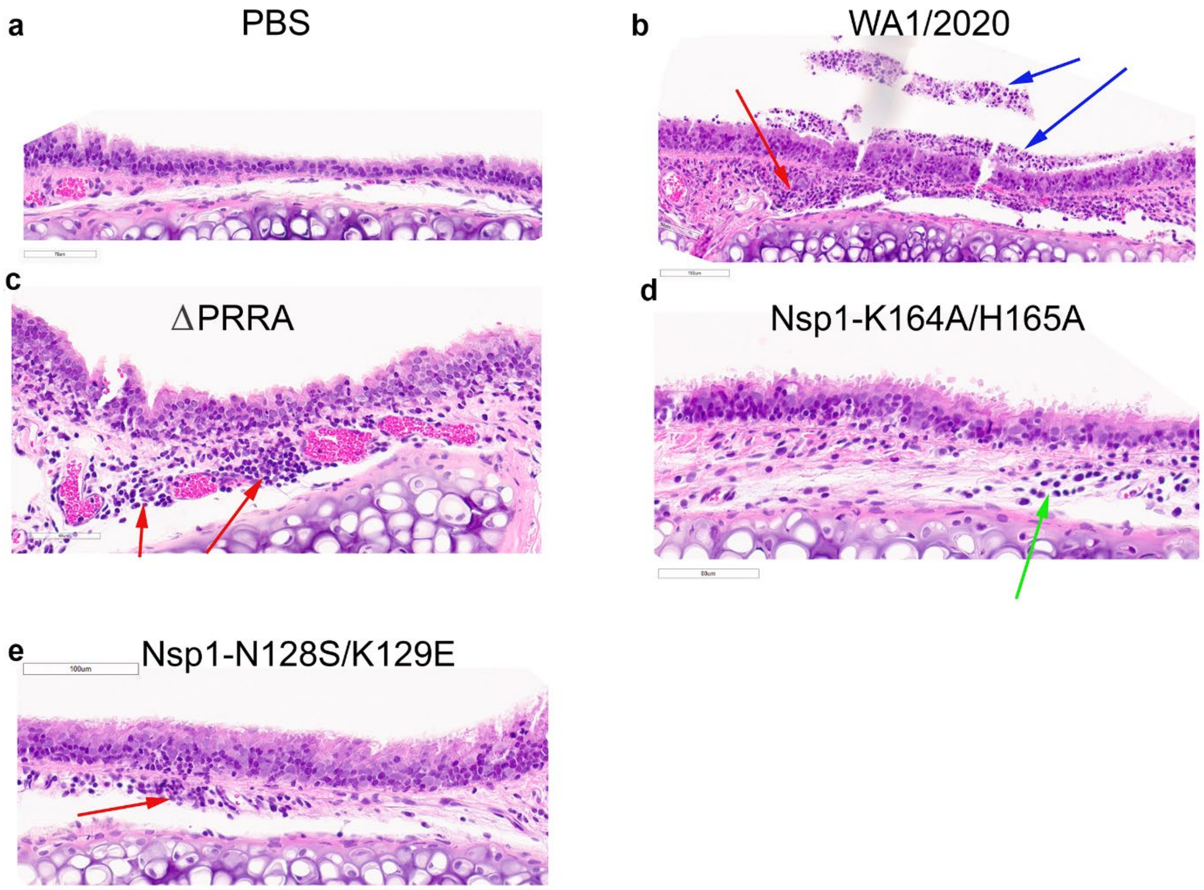

Supplementary Fig. 3: Attenuation of Nsp1-K164A/H165A in trachea of Syrian hamsters. **a** normal epithelium of trachea from PBS-treated hamsters. Scale bar, 70 μM. **b** WA1/2020 infected trachea had luminal neutrophilic accumulations (blue arrows) as well as submucosal lymphoplasmacytic and neutrophil infiltrates (red arrow). Scale bar, 100 μM**. c** submucosal lymphoplasmacytic infiltrates. Scale bar, 60 μM. **d** a few lymphocytes (indicated by the green arrow) were found in submucosal space. Scale bar, 80 μM. **e** submucosal lymphoplasmacytic infiltrates. Experiments were conducted once, with multiple biological replicates. Scale bar, 100 μM.

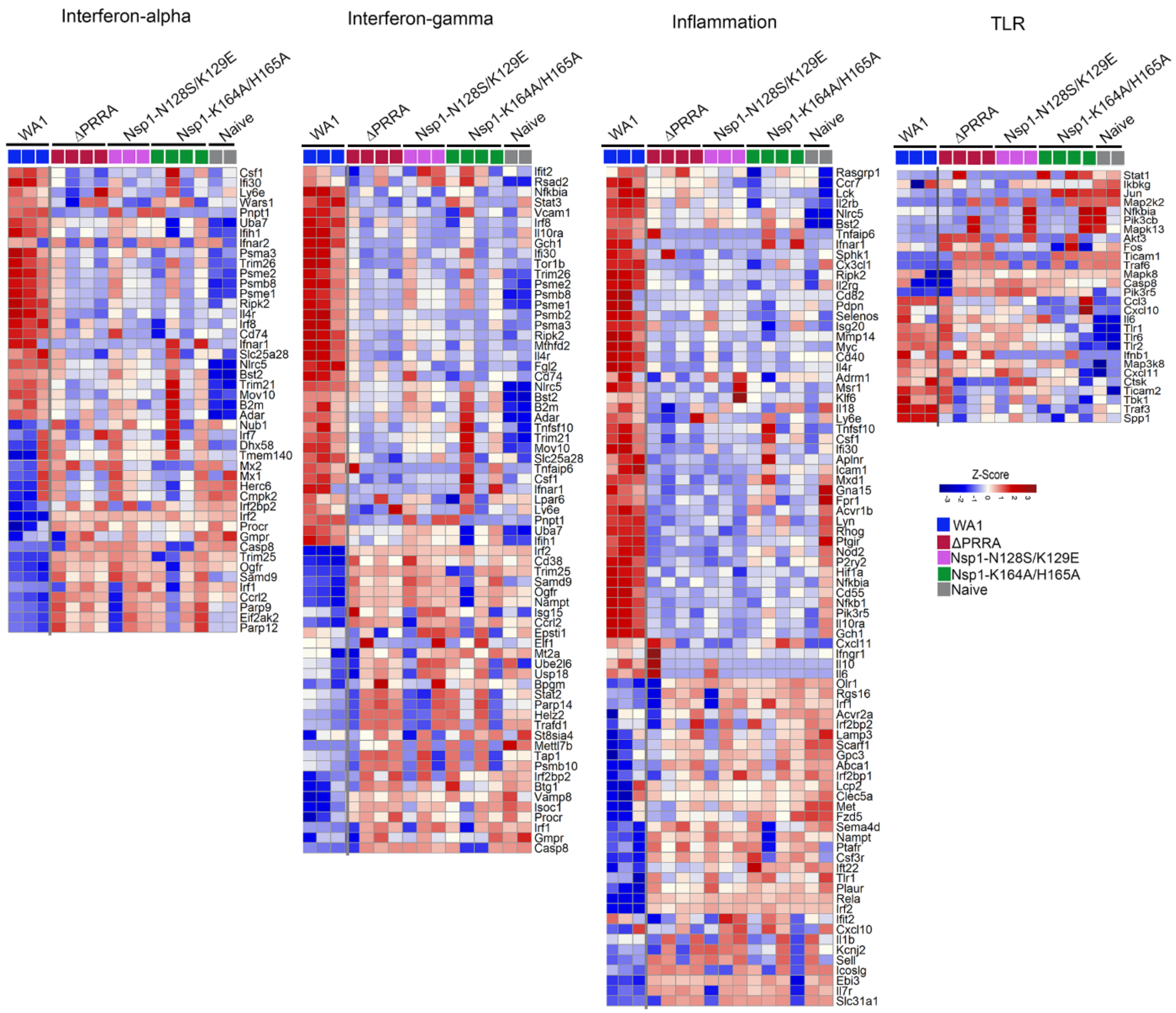

Supplementary Fig. 4: Bulk transcriptomic profiling of hamster lung tissues at day 4 post SARS-CoV-2 infection. Heatmap analysis of interferon and inflammation signalling pathways in nasal turbinates. The data represents the Z-scores derived from FPKM values of RNAseq transcriptomic analysis. Red color indicates positive Z-Score (genes that are upregulated) and blue for negative Z-score (genes that are downregulated).

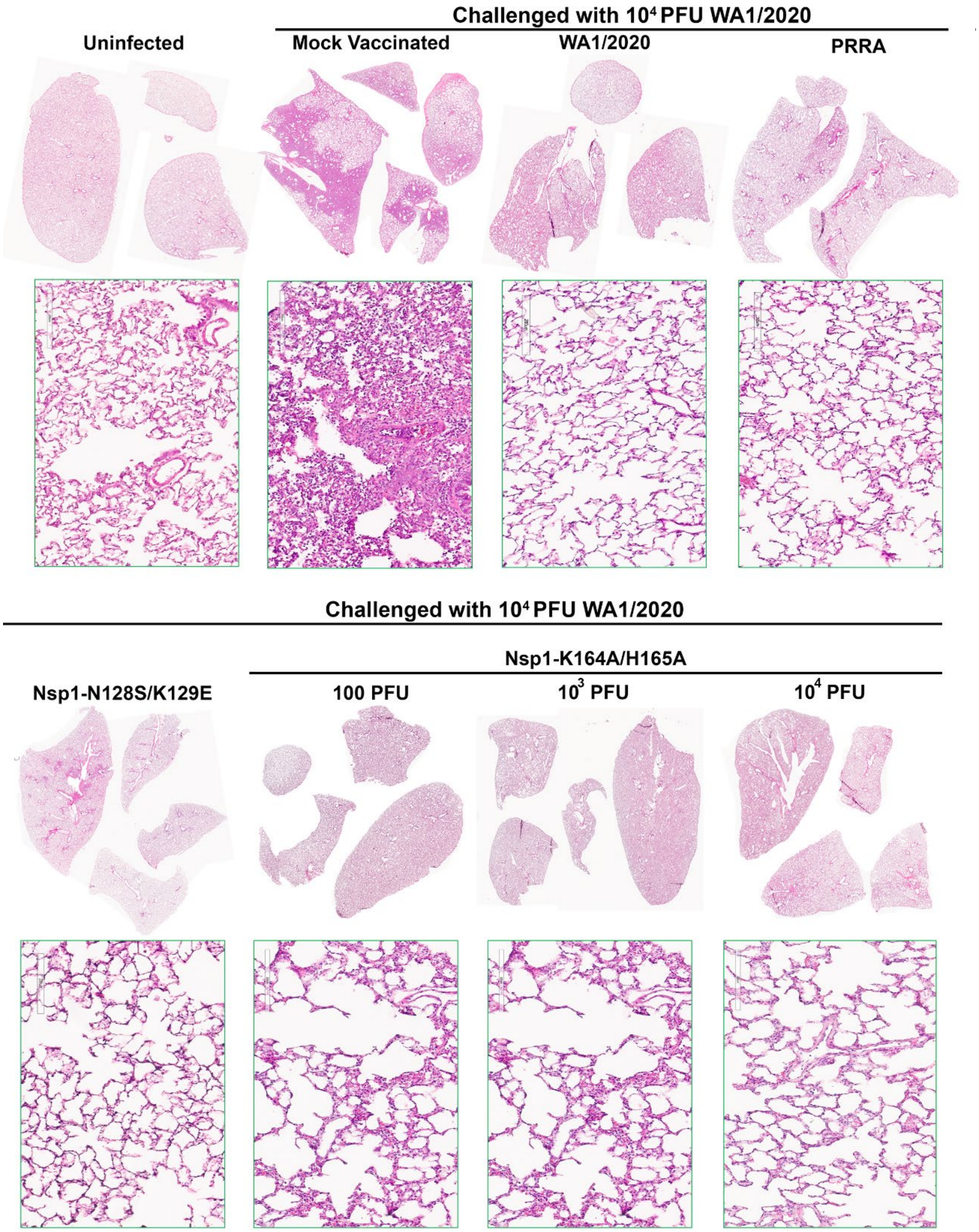

Supplementary Fig. 5: Syrian hamsters immunized with Nsp1-K164A/H165A displayed minimal lung pathology upon WA1/2020 challenge. Representative HE stained images of hamster lungs corresponding to Fig. 6 are presented. An image of multiple lobes as well as an image from the alveolar

space from each group are included. Experiments were conducted once, with multiple biological replicates.

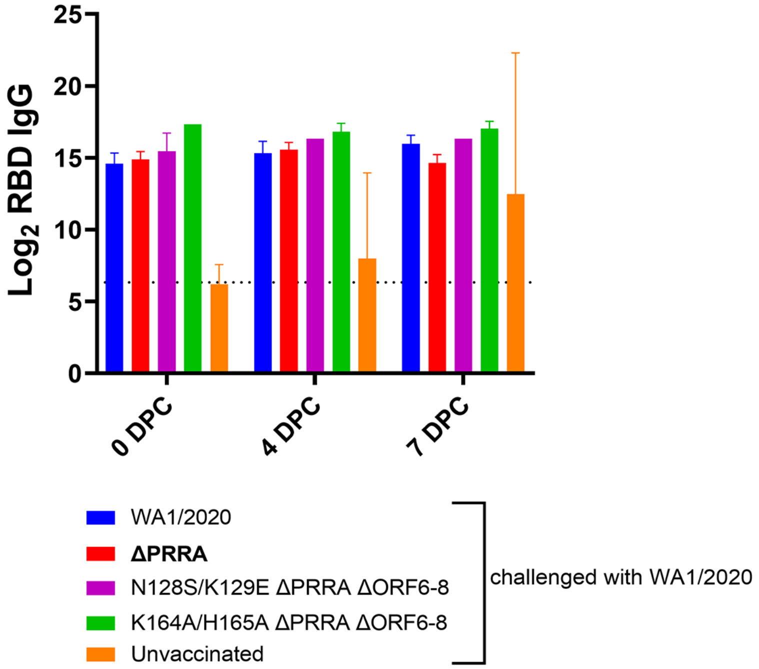

Supplementary Fig. 6: Antibody response of immunized and convalescent hamsters following re-challenge. RBD binding antibody titers were determined by ELISA from serum samples collected from unvaccinated, immunized, and convalescent hamsters at 0 (n=7), 4 (n=4), and 7 (n=3) dpc in a single experiment. At 4 and 7 dpc, RBD IgG titers increased in the unvaccinated group but not in the other groups. Error bars indicate standard deviation.

Supplementary Table 1- List of reagents used in this study

REAGENT or RESOURCE	SOURCE	IDENTIFIER
**Bacterial and Virus Strains**		
SARS-Coronavirus 2, Isolate USA-WA1/2020	BEI	Cat # NR-52281
WA1-ΔPRRA	This paper	Cat # NR-56461
WA1-ΔPRRA-ORF6-8-Nsp1$^{N128S/K129E}$	This paper	
WA1-ΔPRRA-ORF6-8-Nsp1$^{K164A/H165A}$	This paper	
Endura™ Chemically Competent Cells	Lucigen	Cat # 60240-2
TransforMax™ EPI300™ Competent Cells	Lucigen	Cat # C300C105
**Chemicals, Peptides, and Recombinant Proteins**		
Lipofectamine-3000 Transfection Reagent	ThermoFisher	Cat # L3000015
mMESSAGE mMACHINE™ T7 Transcription Kit	ThermoFisher	Cat # AM1344
Luciferase Assay System	Promega	Cat # E1501
**Experimental Models: Cell Lines**		
Lenti-X™ 293T Cell Line	Takara	Cat # 632180
EpiAirway cells	MatTek	AIR-100-HCF
A549-hACE2	BEI	Cat # NR-53821
Vero (C1008) E6	ATCC	Cat # CRL-1586
**Oligonucleotides**		
Primer M13 F (GTAAAACGACGGCCAGT)	Facility for Biotechnology Resources, FDA	
Primer N128S/K129Ef (TAAGAACGGTAGTGAGGGAGCTGGTGGCCATAGTTA)	Facility for Biotechnology Resources, FDA	
Primer N128S/K129E r (CACCAGCTCCCTCACTACCGTTCTTACGAAGAAGAA)	Facility for Biotechnology Resources, FDA	
Primer K164A/H165Af (AAAACTGGAACACTGCCGCCAGCAGTGGTGTTACCCGTGA)	Facility for Biotechnology Resources, FDA	
Primer K164A/H165Ar (GGGTAACACCACTGCTGGCGGCAGTGTTCCAGTTTTCTTGAA)	Facility for Biotechnology Resources, FDA	
Primer NheIr (CACGAGCAGCCTCTGATGCA)	Facility for Biotechnology	

	Resources, FDA	
Primer ΔPRRA-f (ACTCAGACTAATTCTCGTAGTGTAGCTAGTCAATC)	Facility for Biotechnology Resources, FDA	
Primer ΔPRRA-r (ACTAGCTACACTACGAGAATTAGTCTGAGTCTGAT)	Facility for Biotechnology Resources, FDA	
Primer Bgl II r (CAGCATCTGCAAGTGTCACT)	Facility for Biotechnology Resources, FDA	
Primer Mf (TTAATTTTAGCCATGGCAGA)	Facility for Biotechnology Resources, FDA	
Primer ORF68f (TTTGCTTGTACAGTAAACGAACAAACTAAAATGTC)	Facility for Biotechnology Resources, FDA	
Primer ORF68r (TTTTAGTTTGTTCGTTTACTGTACAAGCAAAGCAA)	Facility for Biotechnology Resources, FDA	
Primer AvrIIr (GAAGTCCAGCTTCTGGCCCA)	Facility for Biotechnology Resources, FDA	
**Recombinant DNA**		
psPAX2	Addgene	Cat # 12260
pcDNA-SARS-CoV-2 Spike	Liu et al., 2021	
pTRIP-luc	Liu et al., 2010	
**RNAscope Reagents**		
RNAscope 2.5 HD RED kit	ACD	Cat #322373
V-nCoV2019-orf1ab	ACD	Cat #895661
Mm PPIB probe (positive control)	ACD	Cat #313911
dapB probe (negative control)	ACD	Cat #310043
**Software and Algorithms**		
Prism 9.0 software	GraphPad	
SnapGene	GSL Biotech LLC	
