# Supplementary material for: Intranasal delivery of a rationally attenuated SARS-CoV-2 is immunogenic and protective in Syrian hamsters
Source: Nat Commun. 2022 Nov 10;13:6792. doi: 10.1038/s41467-022-34571-4 (PMC9648440; doi:10.1038/s41467-022-34571-4)
Supplement: Supplementary file 3 — Reporting Summary [file 41467_2022_34571_MOESM3_ESM.pdf]

nature portfolio

Corresponding author(s): Tony T. Wang

Last updated by author(s): Oct 18, 2022

Reporting Summary

Nature Portfolio wishes to improve the reproducibility of the work that we publish. This form provides structure for consistency and transparency in reporting. For further information on Nature Portfolio policies, see our Editorial Policies and the Editorial Policy Checklist.

Statistics

For all statistical analyses, confirm that the following items are present in the figure legend, table legend, main text, or Methods section.

n/a	Confirmed	
☐	☒	The exact sample size (*n*) for each experimental group/condition, given as a discrete number and unit of measurement
☐	☒	A statement on whether measurements were taken from distinct samples or whether the same sample was measured repeatedly
☐	☒	The statistical test(s) used AND whether they are one- or two-sided<br>*Only common tests should be described solely by name; describe more complex techniques in the Methods section.*
☐	☒	A description of all covariates tested
☒	☐	A description of any assumptions or corrections, such as tests of normality and adjustment for multiple comparisons
☐	☒	A full description of the statistical parameters including central tendency (e.g. means) or other basic estimates (e.g. regression coefficient) AND variation (e.g. standard deviation) or associated estimates of uncertainty (e.g. confidence intervals)
☐	☒	For null hypothesis testing, the test statistic (e.g. *F*, *t*, *r*) with confidence intervals, effect sizes, degrees of freedom and *P* value noted<br>*Give P values as exact values whenever suitable.*
☒	☐	For Bayesian analysis, information on the choice of priors and Markov chain Monte Carlo settings
☒	☐	For hierarchical and complex designs, identification of the appropriate level for tests and full reporting of outcomes
☒	☐	Estimates of effect sizes (e.g. Cohen's *d*, Pearson's *r*), indicating how they were calculated

Our web collection on statistics for biologists contains articles on many of the points above.

Software and code

Policy information about availability of computer code

Data collection	Data were collected using Microsoft Excel (Version 2102, Microsoft Office 365), Agilent Cytation 7 with built-in software, Glomax luminometer (Progema), StepOne Plus thermal cycler with built-in software (Qiagen), NovaSeq 6000 sequencer (Illumina, San Diego, CA), Aperio ImageScope (12.4.3.5008), and a Hamamatsu NanoZoomer 2.0-RS whole-slide digital scanner equipped with a 20x objective and a fluorescence module #L11600.
Data analysis	RNAseq data analysis was done using R Studio 1.4.1106 (http://www.R-project.org). Heatmaps were constructed using heatmap library. The gene list for signaling pathways were obtained from hallmark gene sets in Molecular Signatures Database (MSigDB)60. The figures were assembled in Adobe Photoshop. This work utilized the computational resources of the NIH HPC Biowulf cluster (http://hpc.nih.gov). Immunofluorescence analysis software NDP.view2 was used for image processing (Hamamatsu Photonics, Japan).

For manuscripts utilizing custom algorithms or software that are central to the research but not yet described in published literature, software must be made available to editors and reviewers. We strongly encourage code deposition in a community repository (e.g. GitHub). See the Nature Portfolio guidelines for submitting code & software for further information.

Data

Policy information about availability of data

All manuscripts must include a data availability statement. This statement should provide the following information, where applicable:

- Accession codes, unique identifiers, or web links for publicly available datasets
- A description of any restrictions on data availability
- For clinical datasets or third party data, please ensure that the statement adheres to our policy

The RNA-seq datasets have been submitted to The National Center for Biotechnology Information. Metadata file submitted for GEO submission along with the fastq files are included in Supplementary Table 2. All unique/stable reagents generated in this study are available from the Lead Contact with a completed Materials Transfer Agreement.The sequencing reads for each sample were mapped to the respective reference genomes of Mesocricetus auratus (BCM_Maur_2.0) by Tophat (v2.1.2). Cufflinks (v2.2.1) was then used to assemble transcripts, estimate abundances and test for differential expression. The sequencing and initial data analysis using Qiagen CLC Genomics Workbench (version 21) was performed by FDA Next Generation Sequencing Core Facility. The sequencing and initial data analysis using Qiagen CLC Genomics Workbench (version 21) was performed by FDA Next Generation Sequencing Core Facility. Raw and processed data have been deposited to NCBI (GEO accession number GSE199922s).

Human research participants

Policy information about studies involving human research participants and Sex and Gender in Research.

Reporting on sex and gender	n/a
Population characteristics	n/a
Recruitment	n/a
Ethics oversight	n/a

Note that full information on the approval of the study protocol must also be provided in the manuscript.

Field-specific reporting

Please select the one below that is the best fit for your research. If you are not sure, read the appropriate sections before making your selection.

☒ Life sciences ☐ Behavioural & social sciences ☐ Ecological, evolutionary & environmental sciences

For a reference copy of the document with all sections, see nature.com/documents/nr-reporting-summary-flat.pdf

Life sciences study design

All studies must disclose on these points even when the disclosure is negative.

Sample size	No sample size calculation was performed. For hamster studies, n≥3 was chosen for each experimental group for calculating statistical significance in the study. In most experiments, however, there are at least 4 hamsters per group except for negative controls (uninfected group). For in vitro experiments. there are 2-6 technical replicates for each treatment/group. These sample sizes are chosen in conformity with similar studies in the field.
Data exclusions	No data were excluded from the study.
Replication	In vitro experiments will be repeated twice. Results from mouse or hamsters studies were obtained either from one independent experiment or pooled from two separate experiments with age-matched animals. All attempts at replication were successful.
Randomization	Animals in this study were randomized into different groups for experiments.
Blinding	Investigators were not blinded in data acquisition or sample collection because it is not possible to conduct the BSL-3 experiment without knowing such information. However, after sample collection, only a sample ID was available for subsequent data analyses which prevents bias.

Reporting for specific materials, systems and methods

We require information from authors about some types of materials, experimental systems and methods used in many studies. Here, indicate whether each material, system or method listed is relevant to your study. If you are not sure if a list item applies to your research, read the appropriate section before selecting a response.

Materials & experimental systems

n/a	Involved in the study
☐	☒ Antibodies
☐	☒ Eukaryotic cell lines
☒	☐ Palaeontology and archaeology
☐	☒ Animals and other organisms
☒	☐ Clinical data
☒	☐ Dual use research of concern

Methods

n/a	Involved in the study
☒	☐ ChIP-seq
☒	☐ Flow cytometry
☒	☐ MRI-based neuroimaging

Antibodies

Antibodies used	SARS nucleocapsid protein (1:800, Sino Biologicals, 40143-MM05), prosurfactant protein C (1:200, EMD Millipore, AB3786), Iba1 (1:100, Abcam, ab5076), and RAGE (1:400, Abcam, ab216329). Secondary antibodies include c Alexa Fluor 488 (A-21206) and Alexa Fluor 647 (A-31571, A-21447) (ThermoFisher, Waltham, MA). The HRP-conjugated goat anti-hamster IgG was purchased from Southern Biotech, Birmingham, Alabama(cat No. 6060-05). The Rabbit anti-hamster IgA antibody (sab 3001a, Brookwood Biomedical, Jemison). An HRP-conjugated goat anti-rabbit IgG (4030-05, Southern Biotech, Birmingham, Alabama).
Validation	The applications of these antibodies were validated by the vendors on the manufacturer's website or data provided in the manuscript.

Eukaryotic cell lines

Policy information about cell lines and Sex and Gender in Research

Cell line source(s)	Vero E6 cell line (Cat # CRL-1586) was purchased from American Type Cell Collection (ATCC). A549-hACE2 (NR-53821) cells were obtained from BEI Resources. Lenti-X cell line was purchased from Takarabio (Cat No. 632180).
Authentication	Vendors routinely validate these cell lines using PCR assays with species-specific primers.
Mycoplasma contamination	No mycoplasma contamination as verified by PCR.
Commonly misidentified lines (See ICLAC register)	none.

Animals and other research organisms

Policy information about studies involving animals; ARRIVE guidelines recommended for reporting animal research, and Sex and Gender in Research

Laboratory animals	Female adult K18-hACE2 mice (12 weeks ago) were previously purchased from the Jackson laboratory and maintained at FDA vivarium first before transfering the BSL-3 laboratory and housed at 22 Celsius degree with 60% humidity with normal dark/light cycle. Male outbred Syrian hamsters were previously purchased from Envigo between 9-11 weeks ago and held at FDA vivarium until aged (>4 months). All experiments were performed within the biosafety level 3 (BSL-3) suite on the White Oak campus of the U.S. Food and Drug Administration. The animals were implanted subcutaneously with IPTT-300 transponders (BMDS), randomized, and housed 2 per cage in sealed, individually ventilated rat cages (Allentown). Hamsters were fed irradiated 5P76 (Lab Diet) ad lib, housed on autoclaved aspen chip bedding with reverse osmosis-treated water provided in bottles, and all animals were acclimatized at the BSL3 facility for 4-6 days or more prior to the experiments. The study protocol details were approved by the White Oak Consolidated Animal Care and Use Committee and carried out in accordance with the PHS Policy on Humane Care & Use of Laboratory Animals.
Wild animals	No wild animals were used in this study.
Reporting on sex	Female adult K18-hACE2 mice and male outbred Syrian hamsters were used in this study to represent both genders.
Field-collected samples	No field collected samples were used.
Ethics oversight	The study protocol details (ASP2020-06 and 2020-30) were approved by the White Oak Consolidated Animal Care and Use Committee and carried out in accordance with the PHS Policy on Humane Care & Use of Laboratory Animals.

Note that full information on the approval of the study protocol must also be provided in the manuscript.
